# PhISCS: a combinatorial approach for subperfect tumor phylogeny reconstruction via integrative use of single-cell and bulk sequencing data

**DOI:** 10.1101/gr.234435.118

**Published:** 2019-11

**Authors:** Salem Malikic, Farid Rashidi Mehrabadi, Simone Ciccolella, Md. Khaledur Rahman, Camir Ricketts, Ehsan Haghshenas, Daniel Seidman, Faraz Hach, Iman Hajirasouliha, S. Cenk Sahinalp

**Affiliations:** 1 School of Computing Science, Simon Fraser University, Burnaby, BC V5A 1S6, Canada;; 2 Department of Computer Science, Indiana University, Bloomington, Indiana 47408, USA;; 3 Cancer Data Science Laboratory, National Cancer Institute, National Institutes of Health, Bethesda, Maryland 20892, USA;; 4 Department of Computer Systems and Communication, University of Milano-Bicocca, 20136 Milan, Italy;; 5 Institute for Computational Biomedicine, Weill Cornell Medicine, New York, New York 10065, USA;; 6 Tri-I Computational Biology and Medicine Graduate Program, Cornell University, New York, New York 10065, USA;; 7 Department of Urologic Sciences, University of British Columbia, Vancouver, BC V5Z 1M9, Canada;; 8 Vancouver Prostate Centre, Vancouver, BC V6H 3Z6, Canada;; 9 Department of Physiology and Biophysics, Englander Institute for Precision Medicine, The Meyer Cancer Center, Weill Cornell Medicine, New York, New York 10065, USA

## Abstract

Available computational methods for tumor phylogeny inference via single-cell sequencing (SCS) data typically aim to identify the most likely *perfect phylogeny tree* satisfying the *infinite sites assumption* (ISA). However, the limitations of SCS technologies including frequent allele dropout and variable sequence coverage may prohibit a perfect phylogeny. In addition, ISA violations are commonly observed in tumor phylogenies due to the loss of heterozygosity, deletions, and convergent evolution. In order to address such limitations, we introduce the *optimal subperfect phylogeny problem* which asks to integrate SCS data with matching bulk sequencing data by minimizing a linear combination of potential false negatives (due to allele dropout or variance in sequence coverage), false positives (due to read errors) among mutation calls, and the number of mutations that violate ISA (real or because of incorrect copy number estimation). We then describe a combinatorial formulation to solve this problem which ensures that several lineage constraints imposed by the use of variant allele frequencies (VAFs, derived from bulk sequence data) are satisfied. We express our formulation both in the form of an integer linear program (ILP) and—as a first in tumor phylogeny reconstruction—a Boolean constraint satisfaction problem (CSP) and solve them by leveraging state-of-the-art ILP/CSP solvers. The resulting method, which we name PhISCS, is the first to integrate SCS and bulk sequencing data while accounting for ISA violating mutations. In contrast to the alternative methods, typically based on probabilistic approaches, PhISCS provides a guarantee of optimality in reported solutions. Using simulated and real data sets, we demonstrate that PhISCS is more general and accurate than all available approaches.

The clonal theory of cancer evolution suggests that cancer is an evolutionary disease where multiple distinct cellular populations (i.e., subclones) emerge through successive rounds of mutation and selection. At the time of clinical diagnosis, most tumors are heterogeneous, consisting of multiple subclones harboring different sets of somatic mutations. Increasing evidence suggests that this phenomenon, better known as “intra-tumor heterogeneity” (ITH), has a profound impact on treatment outcomes, and that the existence of treatment-resistant subclones is one of the main causes of treatment failures (Alizadeh et al. 2015). Deciphering intra-tumor heterogeneity and tumor evolutionary history thus represent some of the key challenges in designing effective combinatorial therapies and better understanding of dynamics of cancer initiation and progression.

Most of the existing approaches for studying ITH are based on analyzing data from high-throughput bulk sequencing experiments where only an average signal over a large number of cells is obtained. In the past few years, numerous computational methods for analyzing such signals with the aim of inferring tumor subclonal composition and evolutionary history have been developed (Strino et al. 2013; Hajirasouliha et al. 2014; Jiao et al. 2014; Deshwar et al. 2015; El-Kebir et al. 2015, 2016; Malikic et al. 2015; Popic et al. 2015; Yuan et al. 2015; Marass et al. 2016; Donmez et al. 2017; Satas and Raphael 2017). Even though these methods employ a variety of computational approaches, each with a particular strength, all have theoretical limitations, mainly due to the limited resolution offered by bulk sequencing data, admitting multiple equivalent solutions (e.g., a linear topology for any possible instance of the problem).

While it is still expensive and experimentally challenging to robustly perform single-cell library preparation, recent technological advancements in single-cell sequencing (SCS) potentially provide higher resolution data for studying ITH. Single-cell data sets are, however, characterized by high levels of sequencing noise that includes both false positive (e.g., due to the read errors) and false negative (e.g., due to the allele dropout or variance in sequence coverage) mutation calls, as well as *missing* values for mutations from sites affected by DNA amplification failure. This necessitates the development of sophisticated computational methods that are sensitive to the noise characteristics of SCS data, while incorporating the assumptions of the clonal theory of cancer evolution to tumor evolution modeling.

We note that a final additional source of noise is *doublets*, two (or rarely more) cells with heterogeneous mutation profiles treated as a single cell. There already exist computational tools for identifying and decoupling doublets, in particular Single Cell Genotyper (Roth et al. 2016), which we employ in this study as a preprocessing step for the purpose of reducing their impact in our analysis. Furthermore, a recently developed tool, SiCloneFit (Zafar et al. 2019) offers the ability to identify doublets while resolving ITH through a Gibbs sampling approach.

A number of available methods for studying ITH via the use of SCS data are based on probabilistic approaches with the goal of inferring the *most-likely perfect-phylogeny* for a tumor. SCITE (Jahn et al. 2016), for example, is a Markov chain Monte Carlo (MCMC) search method that aims to infer the maximum-likelihood (ML) mutational history from a potentially incomplete and noisy matrix containing genotypes of single cells. OncoNEM (Ross and Markowetz 2016) is a maximum-likelihood-based search approach to identify homogeneous cellular subpopulations and infer both their genotypes and the tree describing their evolutionary history. For achieving their respective goals, SCITE and OncoNEM both rely on the *infinite sites assumption* (ISA), that is, that each genomic position is affected by at most one mutation hit in the entire tumor phylogeny.

A more recent maximum-likelihood-based approach, SiFit (Zafar et al. 2017), aims to extend the above by employing a model of evolution that accounts for deletions, loss of heterozygosity (LOH), and recurrent point mutations on genomic sites. However, none of the above approaches provide means to integratively use SCS with bulk sequencing data, which, in principle, may provide additional guidance to the tumor phylogeny reconstruction process. Another recent tool, ddClone (Salehi et al. 2017), is the first to combine the strengths of bulk and SCS data in a joint statistical inference model for the most likely tumor subclonal composition. However, ddClone does not aim to build a tumor phylogeny and is not suitable to study cancer evolution. Finally, B-SCITE (Malikic et al. 2019) is a newly developed method with the aim of integrating SCITE with CITUP (Malikic et al. 2015) so as to make joint use of SCS and bulk sequencing data. B-SCITE is an MCMC-based tool and, as per SCITE, it does not account for ISA violations.

Even though the above methods for SCS data analysis are probabilistic, many of the related methods for bulk sequencing data analysis are combinatorial in nature (Strino et al. 2013; Hajirasouliha et al. 2014; El-Kebir et al. 2015, 2016; Malikic et al. 2015; Popic et al. 2015). Combinatorial, in particular integer linear programming (ILP), formulations for phylogeny inference have been available in the literature for a while. One example is the *haplotype inference problem* (HIP) (Gusfield et al. 2007), where given a binary incomplete matrix *M* of *n* rows (corresponding to *species*) and *m* columns (corresponding to *sites*), the goal is to decompose each row to two binary vectors (haplotypes) so that the haplotypes can fit in a *Perfect Phylogeny*, that is, a phylogeny satisfying ISA. HIP can be formulated and efficiently solved as an instance of ILP. Later, a similar formulation was proposed in Gusfield (2015) to solve the *Persistent Phylogeny Problem* (Goldberg et al. 1996; Bonizzoni et al. 2012). A persistent phylogeny is one in which each mutation is allowed to be “lost” at most once. Recently, an extension of the formulation from Gusfield (2015) was proposed in Bonizzoni et al. (2017), where more general phylogeny models are used and the goal is to infer entire cancer phylogenies by the use of bulk sequencing data.

Finally, the notion of *flip distance* was introduced in Ragan (1992) and later explored in Chimani et al. (2010) to compare a matrix *M* (see above) that does not admit a perfect phylogeny with *M*′, a matrix admitting a perfect phylogeny that differs from *M* as little as possible. As will be seen, our method builds on this notion of distance.

ILP formulations for HIP and related problems are routinely solved through commercial tools such as Gurobi or IBM CPLEX, which have been developed over many years and provide reliable and fast solutions for relatively small-sized optimization problems. These solvers aim to optimize a typically linear objective while satisfying a number of linear constraints. As such, ILP is related to another fundamental problem, the *Boolean constraint satisfaction problem* (CSP) that can be used as an alternative for modeling many ILP problems encountered in practice.

Perhaps the best-known variant of CSP is the satisfiability problem (SAT) which asks to find a Boolean assignment to a set of input variables to satisfy (the conjunction of) a number of Boolean constraints. Another variant is Max-SAT, which asks to find a Boolean assignment to variables so that not necessarily all but the maximum number of input constraints are satisfied. Finally, the weighted version of Max-SAT, which can be abbreviated as wMax-SAT, asks for the assignment that maximizes the sum of (user-defined) weights of the constraints satisfied. The generality of wMax-SAT has prompted the development of many tools to solve them with the goal of obtaining solutions to practical instances of NP-complete problems. These tools compete in the annual SAT conference on several benchmarking data sets generated by a wide variety of applications (see http://www.satcompetition.org). Recently developed wMax-SAT solvers such as MAXINO (Alviano et al. 2015) and MaxHS (Davies and Bacchus 2011, 2013a,b) are very fast; in addition, MaxHS is open source. A number of studies had already demonstrated the utility of CSP solvers for the haplotype inference problem and its variants - before the advent of high-throughput sequencing (Lynce and Marques-Silva 2006; Neigenfind et al. 2008; He et al. 2010). Nevertheless, to the best of our knowledge, no study has explored the use of CSP in the context of intra-tumor heterogeneity or tumor phylogeny modeling.

## Results

In this paper, we introduce three combinatorial formulations for inferring tumor phylogenies via an integrative use of single-cell and bulk sequencing data. (1) Our first formulation generalizes (Chimani et al. 2010) by asking to minimize a weighted sum of potential false negative (which are common) and false positive (which are rare) mutation calls in genotypes of single cells, whose correction will result in a perfect phylogeny. (2) The goal of our more general formulation is to compute a *subperfect* phylogeny, which not only requires such mutation calls to be corrected but also needs the elimination of (at most a user defined number of) mutations that violate ISA (e.g., due to LOH). More specifically, this formulation asks to minimize a weighted sum of mutations to be corrected, given an upper bound on the number of mutations to be eliminated (due to ISA violations) in order to achieve a perfect phylogeny. Note that this problem differs from that of the “maximum character compatibility” problem, which aims to identify the maximum number of mutations for which a perfect phylogeny is possible. (3) Our most general formulation has additional constraints imposed by the use of variant allele frequencies (VAFs) of single nucleotide variants (from regions not affected by copy number aberrations). VAFs of such mutations can be estimated from bulk sequencing data (as a proxy to the cellular prevalence of a given mutation). These *lineage constraints* impose ancestor-descendant dependencies among mutation pairs (e.g., the prevalence of an ancestral mutation cannot be lower than that of a descendant) or triplets (e.g., the prevalence of an ancestral mutation cannot be lower than the sum of two descendant siblings) and improve inference accuracy. Our formulation allows for one or more samples from the same tumor to be bulk-sequenced independently; each such sample introduces additional lineage constraints that need to be satisfied, within a user-defined error tolerance. For this formulation, eliminating “potential ISA violating mutations” is especially important since the cellular prevalence values could be incorrectly estimated. In fact even VAFs could be incorrectly estimated due to undetected copy number alterations. For example, a copy number gain impacting the allele including the mutation would incorrectly increase its VAF and that impacting the other allele would have the opposite effect. The incorrect VAF estimates may contradict the SCS data, leading to incorrect tumor phylogenies. We describe computational solutions to each of the three formulations to address problems of varying complexity and data availability (i.e., some data sets have no ISA violations and some do not come with matching bulk sequencing data).

We name our general formulation and the resulting program PhISCS (Ph*ylogeny of tumors using* I*ntegrated bulk and* S*ingle*-C*ell* S*equencing data*), and offer two options: (1) PhISCS-I expresses our formulation in the form of an ILP and efficiently solves it by the use of the Gurobi Optimizer; and (2) PhISCS-B expresses our formulation in the form of a Boolean CSP and solves it by the use of open source solvers for wMax-SAT such as MaxHS, often more efficiently than PhISCS-I.

Many of the available tools for studying intra-tumor heterogeneity formulate the problem as an ILP or quadratic integer programming (QIP) and solve it via commercial tools such as Gurobi or CPLEX. Our CSP formulation (specifically in wMax-SAT) is the first to express a tumor phylogeny reconstruction problem combinatorially but in a form other than ILP/QIP. Additionally, unlike most of the available alternatives, PhISCS has the ability to integrate single-cell and bulk sequencing data. Furthermore, recent studies suggest that ISA, that forms the basis for most of the above tools, could be violated in tumor phylogenies (Kuipers et al. 2017; Zafar et al. 2017), making it impossible to establish a perfect phylogeny. PhISCS addresses this issue by eliminating (a small number of) mutations that violate ISA or have incorrect VAF estimates, with a cost reflected in the objective, and solves the tumor phylogeny reconstruction problem for both simulated and real data more efficiently and more accurately than the available alternatives, including B-SCITE. (In order to simplify presentation of the model and results, we treat losses of reference allele and copy number gains [of any of the two alleles] as a type of ISA violation.)

Our final contribution is on assessing the (dis)similarity between two tumor phylogenies, typically between *G*, the ground truth tree, and *T*, the tree inferred by any method. Commonly used measures of similarity between tumor phylogenies such as *lineage consistency* and *nonlineage consistency* (used by Malikic et al. 2015 and others), are defined based on the proportion of mutation pairs with the same lineage relationship in the two trees and fail to capture fundamental topological differences between simulated ground truth and inferred trees, especially of different levels of *granularity* (Karpov et al. 2019). Clustering accuracy used by AncesTree (El-Kebir et al. 2015) and coclustering accuracy used by B-SCITE (Malikic et al. 2019) suffer from the same problem as well (Karpov et al. 2019).

An alternative to the above measures used in the phylogenetics literature is the Robinson-Foulds (RF) distance, which can be thought as the number of single edge cut partitionings of one tree that could not be obtained (by a single edge cut) in the other. However, RF distance cannot be robustly applied to tumor phylogeny comparison due to the high false negative rates observed in single-cell sequencing. (The relocation of only one single-cell as a result of the high false negative rate may result in very high RF distance.) As a result, RF distance is not commonly used in tumor evolution studies (with the exception of Zafar et al. 2017); nevertheless, we demonstrate that, even under the RF measure, PhISCS exhibit superior performance to the alternatives.

In order to overcome the limitations of all above measures, it is appealing to use the standard tree edit distance (TED) (Zhang et al. 1992) and its derivatives. Even though TED is NP-hard to compute for unordered trees, there are some (worst-case exponential-time) algorithms that work well in practice for reasonably small trees. However, TED is defined only for trees where each node has a single label. Here, we show how to generalize TED for tumor phylogenies where each node may have more than one label (mutation) but each label is unique to a specific node.

As will be shown, the resulting *tumor phylogeny tree edit distance* measure (TPTED) captures topological (dis)similarities between tumor phylogenies. Additionally, the recently introduced *multilabeled tree dissimilarity* measure, MLTD, is less general than TPTED but has the advantage of being polynomial-time computable (Karpov et al. 2019). Furthermore, it can still capture the differences between tumor phylogenies of different granularities accurately in all our simulations. We also consider its dual, *multilabeled tree similarity measure*, MLTSM, to compare tumor phylogenies.

In what follows, we first demonstrate the comparative performance of PhISCS against available tools on simulated data under various parameter settings. Then, we present an application of PhISCS on two real SCS data sets.

### Results on simulated data

In order to assess PhISCS against alternative methods, we first benchmarked it on simulated data by using three distinct measures of accuracy. As will be seen, PhISCS outperforms all available alternatives on simulated data with respect to all measures of accuracy.

Below, we first provide a running time analysis for PhISCS, primarily for the purpose of identifying its fastest variant. Then, we briefly describe the measures we used to assess PhISCS results in comparison to alternative methods. (A detailed description of these measures as well as how we generate our simulated data can be found in the Supplemental Material.) We finally demonstrate how well PhISCS performs against the alternatives based on these measures using various simulated data.

#### Comparative running time analysis of PhISCS

We start with a running time comparison between PhISCS-B and PhISCS-I. There are a number of available constraint satisfaction software tools that could be used for PhISCS-B. In order to identify the best performing CSP solver, we evaluated the top-performing tools from the 2017 Max-SAT competition (http://mse17.cs.helsinki.fi/index.html) on simulated data for the limited version of the problem where no ISA violations are allowed and no bulk sequencing data used. The competition has both unweighted and weighted tracks. The three top-performing Max-SAT solvers in the weighted competition were MaxHS, QMaxSAT, and MAXINO. In addition, two other available tools, CPLEX/ILOG by IBM Research and Z3 by Microsoft Research have been benchmarked by the competition organizers. Among these tools, CPLEX/ILOG consistently performed the worst in Max-SAT; this was our experience as well. Additionally CPLEX/ILOG is commercial; as a result, we do not present results obtained by using this tool. A comparison of the running times of PhISCS-B implementations using Z3, MaxHS, and MAXINO Max-SAT solvers are provided in Table 1. This table also includes the results by the top-performing ILP solver, that is, Gurobi, for PhISCS-I.

**Table 1.**
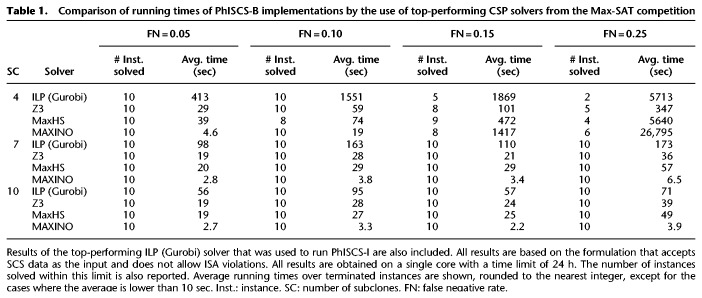
Comparison of running times of PhISCS-B implementations by the use of top-performing CSP solvers from the Max-SAT competition

As demonstrated in Table 1, PhISCS-B outperforms PhISCS-I with respect to running time in all cases. Among the Max-SAT solvers we tested, MAXINO performed the best, typically terminating in a few seconds. The only exceptions are instances with four subclones and higher false negative error rates. However, even in these computationally difficult cases, MAXINO terminates (within a given time limit) on more instances than the other tools (with one exception), which in part explains its higher average running time compared to the other tools (as the running time average was taken over terminated instances). While the average running time of Z3 and MaxHS is higher in comparison to MAXINO, they still perform significantly better than the Gurobi implementation of PhISCS-I. Overall, this demonstrates that PhISCS-B provides a viable alternative to PhISCS-I as well as other existing tumor phylogeny inference approaches that are based on ILP solvers, not only because it is faster but also because it is based on open source Max-SAT solvers rather than commercial ILP solvers.

#### Measuring accuracy in tree inference

In order to assess the (dis)similarity between a simulated ground truth tree, *G*, and the tree inferred by any one of the methods, *T*, we employ three measures, each with distinct properties. Perhaps the natural way to compare tumor phylogenies is through the use of “generalized” tree edit distance. Given two rooted, unordered, and node-labeled trees, TED is defined as the minimum number of *edit operations* (insertion, deletion, and substitution of nodes, and thus their *labels*) to transform one tree to the other (Zhang et al. 1992; Zhang 1993). Although TED is a natural measure to assess the similarity between two tumor phylogenies, it is NP-hard to compute. Furthermore, it is defined for trees where each node has a single label. Below we show how to extend the notion of TED in two distinct ways for measuring tumor phylogeny (dis)similarity.

##### Multilabeled tree similarity measure

In order to address the intractability of TED, a new MLTD measure modifies TED for *multilabeled trees*, where each node may have more than one label but each label is unique to a particular node, as per the tumor phylogenies satisfying ISA (Karpov et al. 2019). Specifically, MLTD is defined as the minimum number of *label* (i.e., mutation) deletions, in addition to an arbitrary number of empty leaf deletions and vertex expansions, applied in any order to transform each of the two trees to reach a maximum size common tree. MLTD is computable in polynomial time and admits efficient practical implementations. In this paper, we use its “dual,” the multilabeled tree similarity measure, which is defined as the size of the maximum common tree of the two tumor phylogenies. To facilitate interpretation of the results, each of the values of MLTSM shown in the figures is normalized by dividing it by the larger number of mutations present in the two trees compared.

##### Tumor phylogeny tree edit distance

Even though TED is NP-hard to compute, there are some algorithms (with worst case exponential running time) with good performance on small trees encountered in practice, for example, in RNA-structure comparison (Mori et al. 2012). These algorithms can be used to compare PhISCS results with the simulated ground truth trees since PhISCS with (bulk sequencing data) produces trees with a single label per node. Note that there are a number of methods that can produce phylogenies with more than one label per node; as a result, it is not always possible to use TED for tumor phylogeny comparison. Thus, we extended TED to a meta-tree edit distance measure that we call tumor phylogeny tree edit distance (see Supplemental Material for a description).

TED/TPTED differs from MLTD in the following key issue: Given two tumor phylogenies *T* and *G*, MLTD corresponds to the minimum number of label deletions (and implied node contractions) in *T*,*G*, so that what remains from them are identical trees. TED/TPTED, on the other hand, is the minimum number of *leaf* or *internal node* label deletions to achieve the same. Naturally TED(T,G),TPTED(T,G)≤MLTD(T,G). Any permutations of edit operations to “convert” *T* to *G* within the realm of MLTD(*T*,*G*) are valid for TED(T,G)/TPTED(T,G), but some permutations of edit operations within the realm of TED(T,G)/TPTED(T,G) would not be valid for MLTD(*T*,*G*).

##### Robinson-Foulds distance

One of the methods that we compare PhISCS against is SiFit, which reports cell lineage trees. In order to compare the two methods, we use the RF distance used by the original SiFit publication by Zafar et al. (2017).

#### Comparing the accuracy of PhISCS and alternative methods

We compared PhISCS against three published methods for single-cell data analysis, namely SiFit, SCITE, and B-SCITE. We were not able to compare PhISCS against OncoNEM since on most input matrices it terminated with an error. Furthermore, no comparison with ddClone was made since it does not infer tumor phylogenies.

**Figure 1.**
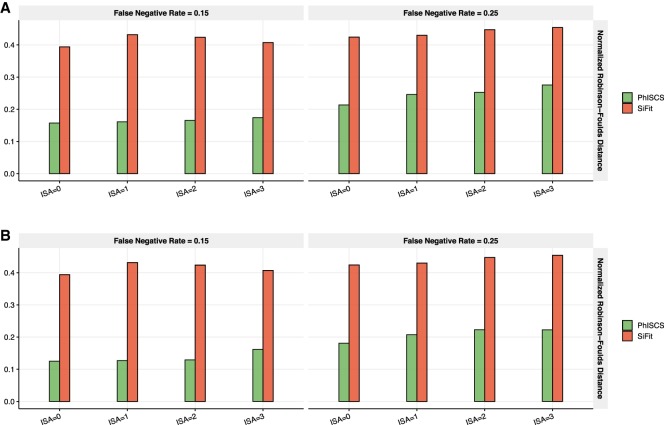
Comparison of PhISCS with SiFit based on the (normalized) Robinson-Foulds distance. (*A*) Results for the case where ISA violations are allowed in PhISCS (SiFit, by default accounts for possible ISA violations), but only SCS data is used as the input. (*B*) Results for the case where ISA violations are allowed in PhISCS and both single-cell and bulk data (with coverage 10,000×) used as its input (SiFit does not support the use of bulk data). In all simulations, 100 single cells were sampled from 10 subclones harboring a total of 40 mutations. Only one bulk sample was used in *B*, potentially limiting the relative advantage of PhISCS by its use of bulk data. Nevertheless, the normalized RF distance between PhISCS inferred tree and the ground truth is reduced by the use of bulk data in all settings. (Note that “ISA” on the *x*-axes denotes the number of simulated ISA violating mutations.)

**Figure 2.**
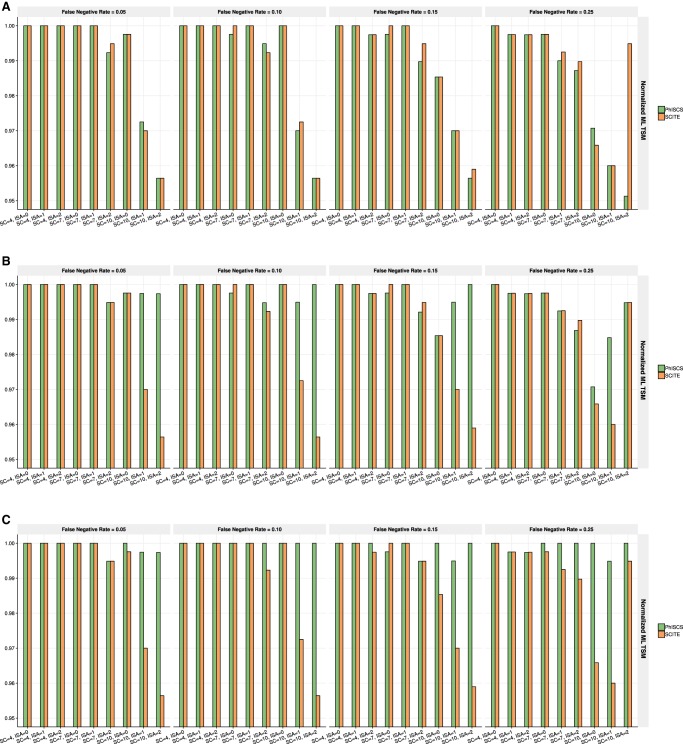
Comparison of PhISCS with SCITE based on normalized MLTSM. (*A*) Normalized MLTSM values when no ISA violations are allowed and no bulk data used in PhISCS. (*B*) Normalized MLTSM values when ISA violations are allowed in PhISCS but bulk data is not part of the input. (*C*) Normalized MLTSM values when PhISCS employs both ISA violations and VAFs derived from the bulk data. In each case, 100 single cells were sampled and the total number of simulated mutations was set to 40. In *C*, one bulk sample was used and coverage of bulk data was set to 10,000×. SC and ISA, respectively, denote the number of subclones and the number of simulated mutations for which ISA is violated. These plots illustrate that PhISCS has comparable performance to SCITE in cases where only SCS data is used; however, the additional use of bulk data substantially improves the performance of PhISCS over SCITE.

**Figure 3.**
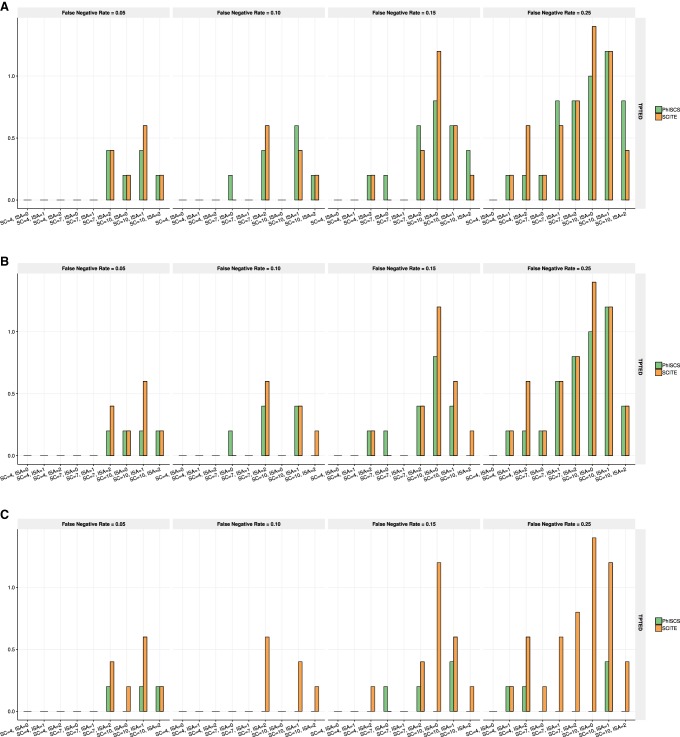
Comparison of PhISCS with SCITE based on TPTED dissimilarity measure. (*A*) TPTED values for SCS data when no ISA violations are allowed. (*B*) TPTED values when ISA violations are allowed in PhISCS. (*C*) TPTED values when ISA violations are allowed in PhISCS and both single-cell and bulk data are used as the input. In each case, 100 single cells were sampled and the total number of simulated mutations set to 40. In *C*, one bulk sample was used and coverage of bulk data was set to 10,000×. SC and ISA, respectively, denote the number of subclones and the number of simulated mutations for which ISA is violated. As our results illustrate, PhISCS has a comparable performance to SCITE in the cases when only SCS data is available; however, the addition of bulk sequencing data improves the performance of PhISCS substantially.

**Figure 4.**
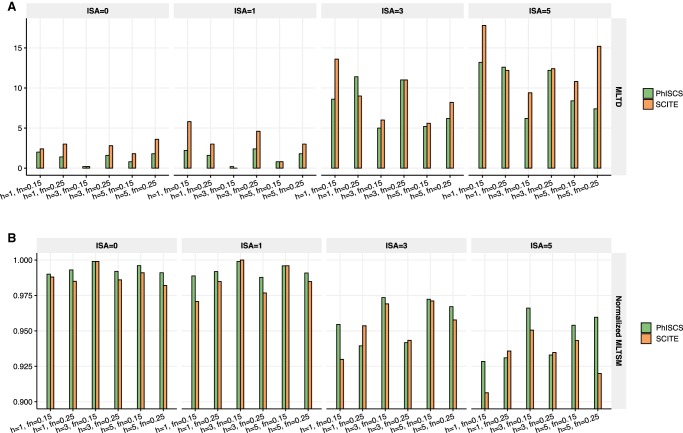
Comparison of PhISCS with SCITE on simulated data with multiple bulk samples and larger number of subclones and mutations. In each case, 10 distinct trees of tumor evolution were generated with 15 subclones and 100 mutations (SNVs). The number of cells was set to 100, while the depth of coverage for bulk data was set to 5000×. *A* compares the two tools with respect to the MLTD dissimilarity measure, while *B* compares them with respect to its dual MLTSM similarity measure (normalized values of MLTSM are shown in the figure). On the *x*-axes, h, fn, and ISA, respectively, denote the number of bulk samples, false negative rate of single-cell data, and the number of simulated mutations for which ISA is violated.

Note that PhISCS-B and PhISCS-I produce highly similar results with the same value of the objective in all cases and slight differences in the resulting genotype corrected matrices *Y*. These slight differences are a consequence of the existence of multiple optimal solutions in some of the cases. All results presented in this section are obtained by taking the average over 10 simulations we generated for each combination of parameters. In all simulations, false positive and missing entries rates were set to 0.0001 and 0.05, respectively. In Figures 1–4 and 6, the minimum subclonal cellular prevalence was set to 5%. For each of the figures, values of other parameters are specified in the caption. We use a similar approach for generating simulated data as in Malikic et al. (2019) (see Supplemental Material for more details).

We first compared PhISCS against SiFit (Zafar et al. 2017). Note that as SiFit reports cell lineage trees, Zafar et al. (2017) used a normalized version of the RF distance as a measure of its accuracy; this was also the measure we used in our comparisons. For enabling this comparison, prior to computing the normalized RF distance, we convert the ground truth tree and tree inferred by PhISCS to cell lineage trees. In doing this conversion, we use the strategy used in Jahn et al. (2016). We present our comparison results between the two methods in Figure 1 which demonstrates that PhISCS outperforms SiFit across all of the simulation settings. These results also indicate that the addition of VAF constraints, derived even from a single bulk sample, can improve tree reconstruction accuracy. Note that the RF distance is not a good measure for comparing tumor phylogenies derived from single-cell genomic sequencing, which suffer from high sequencing noise: Given a ground truth tree with two distinct clones with many cells, the misplacement of only one single-cell in the inferred tree due to noise can result in a very high RF distance.

Next, we present the results of PhISCS with respect to the general tree (dis)similarity measures. Figure 2 demonstrates the performance of PhISCS based on MLTSM in comparison to SCITE. In particular, Figure 2A depicts the results of PhISCS on single-cell data under an infinite sites model. We observe that PhISCS and SCITE have comparable performance in this case. Then, in Figure 2B, we focus on the case with ISA violations allowed in PhISCS but without the use of bulk sequencing data. We observe that the results of PhISCS improve in comparison to the previous case, and overall, it has a slightly better performance compared to SCITE. Finally, when both single-cell and bulk data are available, PhISCS improves its accuracy substantially, as can be seen in Figure 2C, and outperforms SCITE.

Although our MLTD and its dual MLTSM are quickly computed, they may not capture all aspects of topological differences between tumor phylogenies. In Figure 3, we present comparative performance results of PhISCS with respect to the TED/TPTED measure, which is hard to compute but is more general. In particular, Figure 3A presents the results for the case where no bulk sequencing data is available and no ISA violations are allowed when running PhISCS. Results for the case where the same set of data is used as the input, but allowing ISA violations, are provided in Figure 3B. Figure 3C presents the results for the case of integrative use of single-cell and bulk data in PhISCS, while allowing the existence of mutations for which ISA is violated. As can be observed, PhISCS and SCITE have comparable performance for the first case (we suspect that most of the differences are due to MCMC not converging to an optimal solution in SCITE); however, PhISCS significantly benefits from the use of bulk data, outperforming SCITE in all simulations.

**Figure 5.**
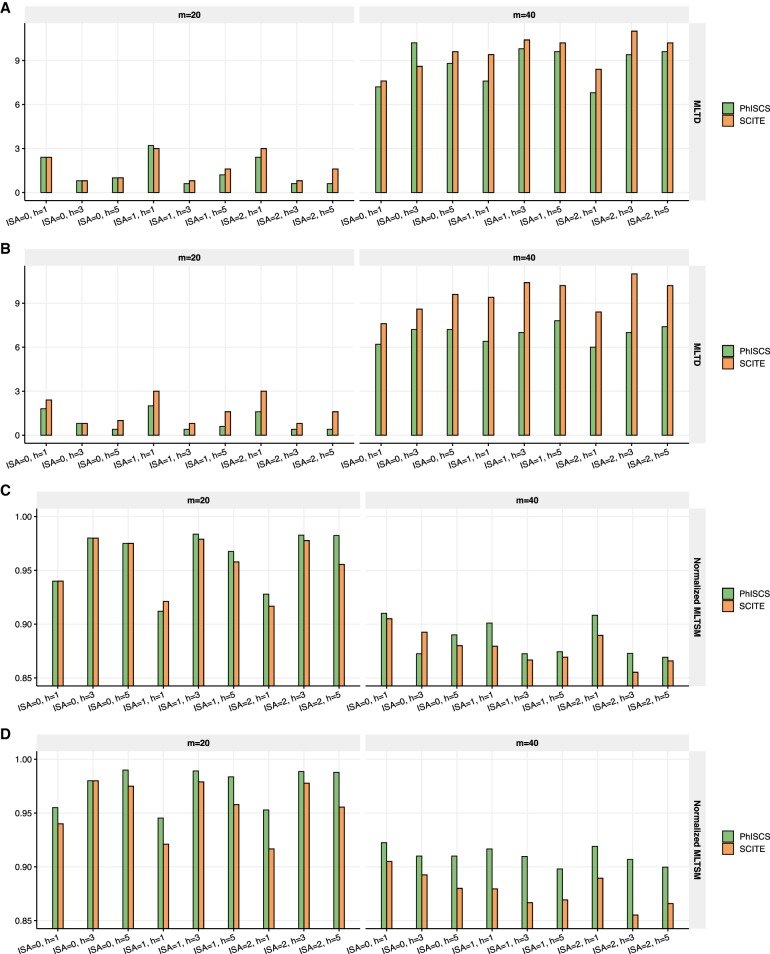
Comparison of PhISCS with SCITE on simulated data where either one or two mutations are acquired at each node (in the latter case, one of the two mutations is an ISA violating mutation). Ten distinct trees of tumor evolution with either 20 mutations (i.e., *s* = 21, *m* = 20) or 40 mutations (i.e., *s* = 41, *m* = 40) were generated and 100 single cells sampled in each case. The false negative rate of single-cell data was set to 0.15. The depth of coverage for bulk data was set to 10,000×, and the minimum cellular prevalence of individual nodes was set to 3% for *m* = 20 and 2% for *m* = 40. *A* and *C*, respectively, show MLTD (dissimilarity) and normalized MLTSM (similarity) measure where ISA violations are allowed, but only SCS data was used as the input. *B* and *D* show, respectively, MLTD (dissimilarity) and normalized MLTSM (similarity) measure where ISA violations are allowed and both single-cell and bulk data are used as the input. On the *x*-axes, h, m, and ISA, respectively, denote the number of bulk samples, the total number of simulated mutations, and the number of simulated mutations for which ISA is violated.

We also compared the performance of PhISCS with SCITE when multiple bulk sequencing samples are available, through the use of both MLTD and its dual MLTSM measures. Results of these comparisons are shown in Figure 4. As can be seen, the performance of PhISCS improves with the increasing number of available bulk samples, suggesting that it can successfully exploit VAF values from multiple bulk samples when such samples are available. Note that these results are based on simulations with a higher number of mutations (100), higher number of subclones (15), higher number of ISA violations (up to 5), and lower bulk sequencing coverage (5000×), also demonstrating that PhISCS accuracy does not deteriorate with increasing scale and complexity of the input data. Similar results are obtained when simulated trees have either one or two mutations gained at each node (in the latter case, at least one of the two mutations must belong to the set of mutations for which ISA is violated). Results of these comparisons are presented in Figure 5.

**Figure 6.**
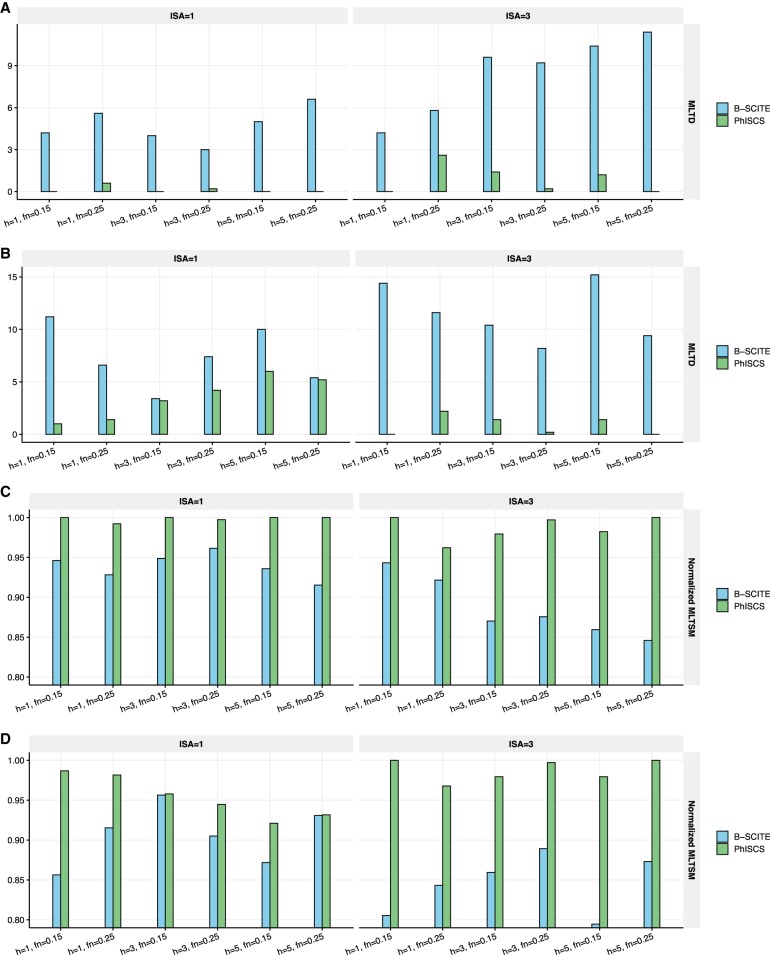
Comparison of PhISCS and B-SCITE according to MLTD and its dual MLTSM similarity measure (normalized values of MLTSM are shown in the figure). In each case, 10 trees of tumor evolution with seven subclones and 40 mutations were simulated and 100 single cells were sampled. Depth of coverage for bulk data was set to 5000×. More details about generating these simulations are provided in the Supplemental Material. *A* and *B* show MLTD (dissimilarity) between inferred and ground truth trees for cases where three SNVs fall into regions having copy number 3 (*A*) or 4 (*B*) in all cancerous cells (i.e., clonal copy number gains 1 or 2 copies of genomic region harboring SNV). *C* and *D* show normalized MLTSM (similarity) between inferred and ground truth trees for cases where three SNVs fall into regions having copy number 3 (*C*) or 4 (*D*) in all cancerous cells. On the *x*-axes, h, fn, and ISA, respectively, denote the number of bulk samples, false negative rate of single-cell data, and the number of simulated mutations for which ISA is violated.

We finally compared PhISCS against B-SCITE, which aims to maximize the joint likelihood of single-cell and (multiple) bulk data samples, and as such, is similar to PhISCS. However, because it is based on an MCMC search as per SCITE, it cannot provide any optimality guarantees. Furthermore, while the bulk sequencing data is used to derive hard constraints for PhISCS, they contribute only to the objective function in B-SCITE. The simulated data we used for comparing PhISCS and B-SCITE (see Supplemental Material) introduce CNA impacted mutations in addition to those that violate ISA. The performance of PhISCS in comparison to B-SCITE is presented in Figure 6. As can be seen, PhISCS is quite robust to copy number alterations, especially in comparison to B-SCITE, and outperforms it in most of the simulation settings.

### Results on real sequencing data

In order to further demonstrate its utility, we applied PhISCS to two real SCS data sets from recent studies. Both data sets provide additional bulk sequencing data with VAF values.

#### Colorectal cancer with liver metastasis

We first tested PhISCS on a CRC2 patient from Leung et al. (2017) (note that in Leung et al. 2017, this patient is sometimes also labeled as CO8). After data preprocessing using the same steps as in B-SCITE (see Supplemental Material; Malikic et al. 2019), we were left with a single-cell data matrix containing mutation profiles of 86 single cells across 25 mutated loci. (See Supplemental Material and, in particular, Supplemental Fig. S1 for results of PhISCS on this data set when no such filtering is performed.)

In the original study (Leung et al. 2017), detailed copy number profiling of primary and metastatic tumor samples was performed, revealing that most of the mutated loci are nondiploid and fall into genomic regions affected by copy number gains. For this reason, we opted not to use VAF-derived constraints and to run PhISCS using only SCS data. An observation to be made here is that copy number gains are usually not expected to be very challenging for the single-cell data-only version of PhISCS. Namely, the presence/absence status of mutation in a cell is not affected by copy number gains overlapping with the mutation's locus. In fact, if a gain results in an increased number of copies of a variant allele, this can even be potentially beneficial and reduce allelic dropout (since such an event would provide more starting material for the PCR amplification step that is typically performed prior to DNA sequencing).

**Figure 7.**
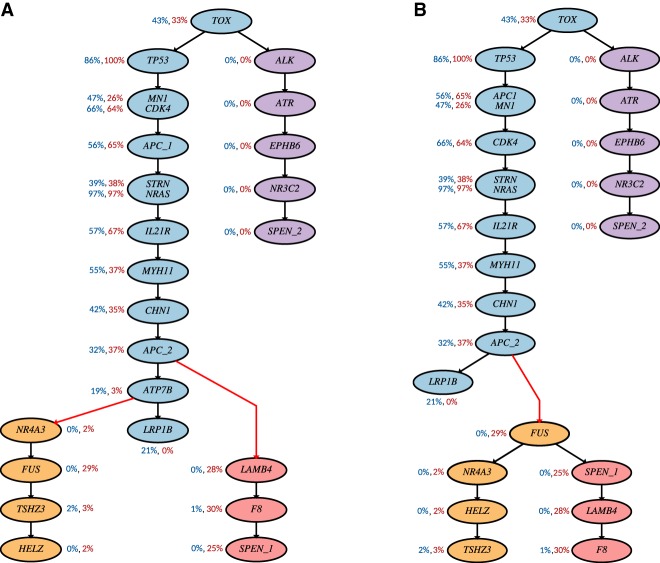
Inferred tumor phylogeny for CRC2 (CO8) patient from Leung et al. (2017) by PhISCS through the use of single-cell data only: (*A*) without allowing mutation elimination; (*B*) with allowing mutation elimination. For each mutation, VAFs derived from bulk data are provided next to the gene label. The first number (colored in blue) represents VAF in the primary tumor and the second number (colored in red) represents VAF in the metastasis. Genes *SPEN* and *APC* harbor multiple mutations. *SPEN_1* denotes mutation at position Chr 1, 16,258,997; *SPEN_2*, mutation at Chr 1, 16,202,934; *APC_1*, mutation at Chr 5, 112,164,646; and *APC_2*, mutation at Chr 5, 112,175,328. Coloring of nodes in *A* is motivated by the coloring used in Figure 6 in Leung et al. (2017). In this coloring, blue and purple nodes represent mutations specific to the primary site, whereas nodes colored in pink and orange represent mutations specific to two distinct metastases. Red edges represent metastatic seeding events. Coloring in *B* is equivalent to the coloring in *A*.

We ran PhISCS using single-cell data, first without considering any ISA violations (the output shown in Fig. 7A), and then allowing ISA violating mutation elimination (the output shown in Fig. 7B). The latter reported a solution eliminating mutation in gene *ATP7B*.

After completing the above analysis, we obtained VAFs from the matching bulk data for both primary and metastasis samples. The average coverage of bulk data at the sites of interest is 100.92× (primary) and 110.60× (metastasis). As shown in Figure 7A, several mutations have one or both VAFs above 0.50. In addition, there are also many pairs of mutations where mutation with lower VAF is placed above the mutation with higher VAF. Both of these are expected and can in most cases be attributed to the presence of copy number aberrations present in the data or variance due to the depth of sequencing. One major exception is mutations in genes *ATP7B* and *NR4A3* and their placement with respect to the mutation in *FUS*. Namely, in the metastatic sample, mutations in *ATP7B* and *NR4A3* have VAFs of 0.03 and 0.02, which is significantly lower than 0.29, that is, the VAF of mutation in *FUS*, which is placed below these two genes in the tree shown in Figure 7A. While, in theory, these, as well as other similar discrepancies, can be explained by copy number events (e.g., a massive gain of variant allele in *FUS* in the metastatic sample), we do not see evidence for the existence of such events in bulk data read counts (where coverage of these mutations is in the range from 129 to 223) or in Figure 5D in Leung et al. (2017) (where copy number profiling of primary and metastatic tumors was performed, suggesting the existence of three or four copies of regions harboring these three mutations). Furthermore, an identical relative order of these mutations can be found in the original study (Leung et al. 2017), which implies an enormous false positive rate in metastatic cells for the mutation in *FUS*. More precisely, out of 20 single cells (all metastatic) where presence of mutation was reported, 10 needed to be altered 1 → 0 during tumor phylogeny inference, implying a false positive rate of 50% for this mutation. Such a high false positive rate cannot be attributed to sequencing artifacts as no false positive for this mutated position was reported in any of the 137 primary cells sequenced. For some of the mutations, the false positive rate reported by SCITE is even higher (e.g., mutation in gene *PTPRD* was originally reported in 13 single cells, and 10 of these mutation calls were estimated as false positives in the solution inferred by SCITE). Additionally, SCITE infers a tumor phylogeny nearly identical (with a minor difference) to the one discussed above.

On the other hand, the tree inferred by PhISCS while allowing ISA violations (Fig. 7B), even though the bulk sequencing data and thus VAF values were not used, includes no VAF inconsistencies that cannot be explained by the observed copy number events and/or variance in VAF values due to depth of coverage. Furthermore, this solution implies no mutation loci with elevated false positive mutation calls, and the substantial decrease in the false positive calls are not compensated by any increase in the false negative calls.

From a biological perspective, looking into the timing of metastatic seeding, the solution reported in Figure 7A suggests two distinct metastatic seeding events: one occurring via cells leaving the primary site at the node labeled with *APC_2* and seeding the metastasis-specific subtree rooted at *LAMB4*, and another occurring when cells from the node labeled with *ATP7B* leave the primary site and seed the metastasis-specific subtree rooted at *NR4A3*. In contrast, the tree reported in Figure 7B requires only a single metastatic seeding event that introduces the metastasis-specific subtree rooted at *FUS*. Here, by metastasis-specific subtree, we refer to the subtree consisting solely of mutations present only in the metastatic sample. Note that the nonzero VAF value in the primary sample for mutations *TSHZ3* and *F8* provides some signal for the possible presence of these mutations in this sample. However, this signal is very likely a consequence of sequencing noise as, for each of the two mutations, there is only a single read supporting the variant allele. In addition, these mutations were not reported in any of the sequenced single cells extracted from the primary tumor.

In conclusion, in comparison to the results obtained assuming the infinite sites model, the tree inferred by PhISCS by allowing ISA violating mutation elimination, better explains both single-cell and bulk data, while requiring only a single metastatic seeding event, demonstrating the importance of this feature of PhISCS in tumor phylogeny reconstruction.

#### Childhood acute lymphoblastic leukemia

**Figure 8.**
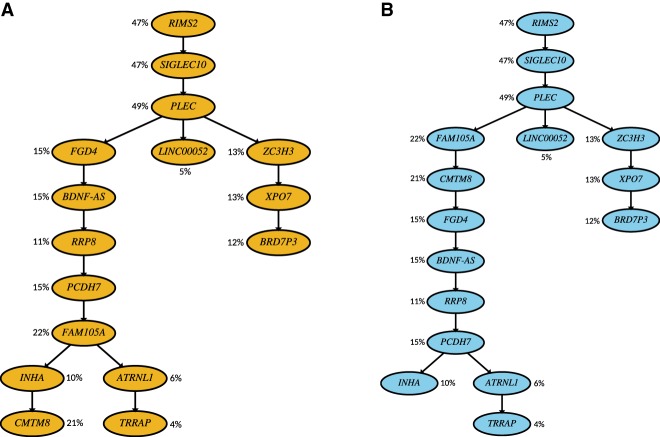
Inferred tumor phylogeny for Patient 2 from Gawad et al. (2014) with (*A*) SCITE and (*B*) B-SCITE. The coloring of the trees is motivated by the colors used for SCITE and B-SCITE in the previous figures. B-SCITE's use of VAFs alter the tree topology by moving the mutation in *CMTM8* higher up in the tree. B-SCITE also reorders the mutations in genes in the linear topology identified by SCITE rooted at *FGD4* —primarily according to their VAFs.

The second data set that we tested PhISCS on is obtained from an acute lymphoblastic leukemia (ALL) study where both single-cell and bulk sequencing data are available (Gawad et al. 2014). We focused on the second patient from this study for which single-cell data provides a strong signal for the existence of multiple subclones with a nonlinear tree topology. The single-cell data matrix used to run PhISCS consists of 16 mutations and 102 single cells (see Supplemental Material). The estimated FN rate for this data set is 0.181749 (Gawad et al. 2014). The trees inferred by SCITE and B-SCITE (Malikic et al. 2019), by the use of SCS data only and by the integrative use of SCS and bulk sequencing data, respectively, with no allowance for ISA violations (see Fig. 8), are generally in agreement with the tree topology published in the original study. However, they differ in the placement of the mutations in genes *RRP8* and *CMTM8*: The VAFs of these mutations do not agree with their placement by SCITE whereas their relative presence/absence in single cells contradicts their placement with B-SCITE.

**Figure 9.**
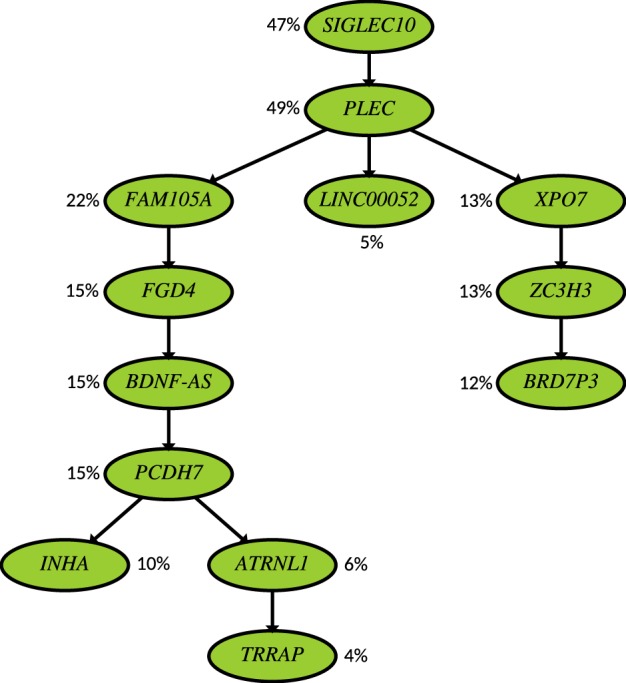
Inferred tumor phylogeny for Patient 2 from Gawad et al. (2014) through the joint use of bulk and SCS data while allowing ISA violations. The topology of the tree and the mutational placements are similar to that of B-SCITE in Figure 8 with the key difference that three mutations are now eliminated by PhISCS: *RRP8* and *CMTM8* are eliminated due to inconsistent VAF and SCS data; *CMTM8* has a similar SCS profile to *INHA* but has a significantly higher VAF. It also has a higher VAF than *RRP8* but appears in 20 fewer cells. In addition, the mutation in *RIMS2* is eliminated by PhISCS as per the figure. Evidence for ISA violations in this gene has been reported earlier (Kuipers et al. 2017).

The results of PhISCS on this data set, using VAFs and allowing the elimination of mutations, are presented in Figure 9. In this topology, the VAF values do not present a contradiction, a highly desirable feature when the VAF values are measured reasonably accurately. For example, the use of VAFs by PhISCS places the mutation in *FGD4* higher in the tree, as it should be. In addition, PhISCS's ability to eliminate mutations with contradictory VAF values and presence in SCS data removes the inconsistencies in the tumor phylogeny. For example, *CMTM8* has a mutation with a high VAF value that contradicts its lower subclonal placement by SCITE in Figure 8. However, in comparison to *RRP8*, it appears in 20 fewer single cells (out of 38). The discrepancies between the VAFs of mutations in *CMTM8* and *RRP8* and fractions of single cells harboring these mutations are possibly a result of an undetected copy number gain on the nonvariant allele for *RRP8* or another undetected copy number gain on the variant allele for *CMTM8*; such copy number gains would result in a lower VAF estimate for *RRP8* or, respectively, a higher one for *CMTM8*. The above observations point out that PhISCS's ability to identify mutations that have been subject to undetected CNAs, combined with its ability to integrate VAFs with SCS data, can help identify previously underexplored copy number aberrations (especially through targeted platforms, where CNA calls could be unreliable) or notice inconsistencies in CNV calls.

## Discussion

PhISCS is a novel combinatorial approach for inferring tumor phylogenies via an integrative use of single-cell and bulk sequencing data. PhISCS offers two options: PhISCS-I expresses our formulation in the form of an ILP and solves it via the Gurobi Optimizer, and PhISCS-B expresses it in the form of a Boolean CSP and solves it via open source wMax-SAT solvers such as MaxHS, often more efficiently than PhISCS-I. (As such, PhISCS-B is the first to express the combinatorial tumor phylogeny reconstruction problem in a form other than ILP/QIP.) PhISCS is the first method for tumor phylogeny inference by integrated analysis of single-cell and bulk sequencing data while allowing ISA violations. Based on three measures of tumor phylogeny (dis)similarity between the ground truth and inferred tree, we have shown by simulations and real data that PhISCS is not only very efficient but is also more accurate than the available alternatives.

One limitation of PhISCS is that it assumes doublets have been eliminated in a preprocessing step; since the doublet rate is being reduced significantly through newer technologies and improved library preparation, we do not expect this to be an important limitation, especially in the analysis of newly generated data.

## Methods

We start describing the algorithmic underpinnings of PhISCS by formulating integrative tumor phylogeny reconstruction as a combinatorial optimization problem. We first focus on two special cases of the problem for the instance in which only single-cell sequencing data is available: (1) a special case where the ISA cannot be violated; and (2) the case where ISA can be violated. We then describe the general integrative problem where both bulk and SCS data are available. We present solutions for this problem in the form of a novel integer linear program as well as a constraint satisfaction problem (CSP).

### PhISCS-I for tumor phylogeny inference via SCS data with no ISA violations allowed

SCS data input to PhISCS is given in a form of ternary matrix *I* with *n* rows and *m* columns, where columns represent mutations and rows represent genotypes of single cells observed in a single-cell sequencing experiment. For a given entry, *I*(*i*, *j*) = 0 indicates the absence, *I*(*i*, *j*) = 1 indicates the presence, and *I*(*i*, *j*) = ? indicates the lack of knowledge about absence or presence (i.e., missing entry) of a mutation *j* in a cell *i*.

We ask to find the minimum weighted number of *bit flips* (typically from 0 to 1 and rarely from 1 to 0) and *bit assignments* (assigning a 0 or 1 to an entry with value ?), where *bit assignments* are not a part of the objective, such that the resulting matrix provides a Perfect Phylogeny (PP). We recall that a binary matrix is a PP if the *three-gametes rule* holds, that is, for any given pair of columns (mutations), there are no three rows (cells) with configuration (1, 0), (0, 1), and (1, 1). *Bit flipping* in the input matrix *I* is essential to building a PP, as some mutation inferences in *I* are false positives and some mutations are not indicated in *I* (false negatives), as they do not have sufficient read support in sequenced single cells. We name any pair of mutations and triplet of cells violating the three-gametes rule as a *conflict* and refer to the PP matrix also as a *conflict-free matrix*.

To allow correction of noisy genotypes in *I* (i.e., bit flips and bit assignments), for each cell *i* and mutation *j*, we introduce a binary variable *Y*(*i*, *j*) which denotes the (unknown) true status (i.e., absence or presence) of the mutation *j* in the cell *i*. If *α* and *β*, respectively, denote false positive and false negative error rates of single-cell data, we have

(1) $$\begin{array}{ll} P(I(i,j)=0|Y(i,j)=0)=(1-\alpha ) & P(I(i,j)=0|Y(i,j)=1)=\text{β}\\ P(I\left(i,j\right)=1|Y\left(i,j\right)=0)=\alpha & P(I\left(i,j\right)=1|Y\left(i,j\right)=1)=\left(1-\text{β}\right) \end{array}.$$

Assuming that the mutated loci are independent and that the missing entries in *I* are noninformative (i.e., bit assignments are not part of the objective), we define the likelihood of an arbitrary conflict-free matrix *Y* as

(2) $$P(I|Y)=\underset{(i,j)\in S}{\prod }P(I(i,j)|Y(i,j))$$

where S is the set of all pairs of integers (*i*, *j*) such that 1 ≤ *i* ≤ *n*, 1 ≤ *j* ≤ *m* and *I*(*i*, *j*) ∈ {0, 1}.

Here, our goal is to find a conflict-free matrix *Y* such that the likelihood *P*(*I*|*Y*) defined in Equation 2 is maximized.

Now, observe that Equation 1 can be rewritten as

(3) $$\begin{array}{rl} & P(I(i,j)=0|Y(i,j))={\left(1-\alpha \right)}^{1-Y(i,j)}\cdot {\text{β}}^{Y(i,j)}=(1-\alpha )\cdot {\left(\frac{\text{β}}{1-\alpha }\right)}^{Y(i,j)},\\ & P(I(i,j)=1|Y(i,j))=\alpha^{1-Y(i,j)}\cdot {\left(1-\text{β}\right)}^{Y(i,j)}=\alpha \cdot {\left(\frac{1-\text{β}}{\alpha }\right)}^{Y(i,j)}, \end{array}$$

and our objective is equivalent to maximizing the logarithm of *P*(*I*|*Y*)_,_ which can be expressed as

(4) $$\underset{(i,j):I(i,j)=0}{\sum }\left[\log(1-\alpha )+\log\frac{\text{β}}{1-\alpha }Y(i,j)\right]+\underset{(i,j):I(i,j)=1}{\sum }\left[\log(\alpha )+\log\frac{1-\text{β}}{\alpha }Y(i,j)\right].$$

In order to enforce that matrix *Y* satisfies the three-gametes rule, for each pair of mutations (*p*, *q*), we first introduce variables *B*(*p*, *q*, *a*, *b*), for each (*a*, *b*) ∈ {(0, 1), (1, 0), (1, 1)}. The variable *B*(*p*, *q*, *a*, *b*) is set to 1 if there exists row *r* such that *Y*(*r*, *p*) = *a* and *Y*(*r*, *q*) = *b*. This property of matrix *B* is guaranteed by adding the following constraints for all 1 ≤ *i* ≤ *n* and 1 ≤ *p*, *q* ≤ *m*:

(5) Y(i,p)+Y(i,q)−B(p,q,1,1)≤1

(6) −Y(i,p)+Y(i,q)−B(p,q,0,1)≤0

(7) Y(i,p)−Y(i,q)−B(p,q,1,0)≤0.

Now, adding constraints

(8) B(p,q,0,1)+B(p,q,1,0)+B(p,q,1,1)≤2

for all 1 ≤ *p*, *q* ≤ *m* suffices to ensure that the three-gametes rule holds for matrix *Y*.

The problem defined above represents an instance of ILP and can be solved using any of the standard ILP solvers.

### Allowing ISA violations in PhISCS-I

As we have already discussed in the Introduction, recent evidence suggests that the ISA might be violated for a subset of mutations in the input data. To account for this, we introduce a more general version of what we discussed in the previous section, where we allow *elimination* (i.e., deletion from the input matrix) of a given (maximum) number of mutations which do not satisfy ISA; the remaining mutations, after genotype corrections, are expected to satisfy PP. In order to achieve this, for each mutation *q*, we introduce binary variable *K*(*q*) which is set to 1 if and only if mutation *q* is among eliminated mutations. To exclude eliminated mutations from the three-gametes rule, we do not require mutational pairs (*p*, *q*), where at least one of *p* and *q* is among eliminated mutations, to fulfill this rule. Therefore, we modify constraint (8) from the integer linear program described above by replacing it with

(9) B(p,q,0,1)+B(p,q,1,0)+B(p,q,1,1)≤2+K(p)+K(q).

The objective defined in Equation 4 is also modified so that the eliminated mutations do not contribute to the objective score. This leads to the following objective to handle the case allowing ISA violations:

(10) $$\underset{(i,j):I(i,j)=0}{\sum }(1-K(j))\cdot \left[\log(1-\alpha )+\log\frac{\text{β}}{1-\alpha }Y(i,j)\right]+\underset{(i,j):I(i,j)=1}{\sum }(1-K(j))\left[\log(\alpha )+\log\frac{1-\text{β}}{\alpha }Y(i,j)\right].$$

All other constraints used previously in the limited version of the problem are preserved. Note that the above objective contains quadratic terms (of the form *K*(*j*)*Y*(*i*, *j*)) which can be transformed to linear variables using standard linearization techniques. One can observe that mutation elimination never decreases data likelihood, hence the global optimum in the above maximization problem is achieved when all variables *K* are set to 1. However, in real applications, we expect only a limited number of ISA violating mutations and therefore set the upper bound *k*_max_ on the number of eliminated mutations, which is implemented by the addition of the following constraint

(11) $$\overset{m}{\underset{q=1}{\sum }}K(q)\leq k_{\max},$$

where *k*_max_ is an empirically estimated constant. It is also possible to computationally estimate *k*_max_ (see Supplemental Material for details).

### Additional ILP constraints to integrate VAFs derived from bulk sequencing data into PhISCS-I

Now, we show how to integrate SCS data with bulk sequencing data—specifically the VAF of each mutation we consider—through additional linear constraints. These constraints will only apply to the set of single nucleotide variants from the regions not affected by copy number aberrations. Suppose that a particular SNV, denoted *M*, satisfies the above requirement; let *v* and *r*, respectively, denote the number of reads supporting the variant and the reference allele at the genomic locus of *M*. The VAF of *M* is typically defined as *v*/(*v* + *r*). Since we are interested in *cellular prevalence* rather than the VAF below, we define vaf(M)=2v/(v+r). (Cellular prevalence represents the expected fraction of cells in the sample that harbor *M*.)

Before defining constraints related to VAFs, we first define the *root node* via a new row, indexed by 0, that represents the genotype of a healthy cell. We also add a new column, indexed by 0, and associated *null mutation M*_0_ which represents mutation specific to the normal cell or, in other words, a germline SNP present in all cells. We set *I*(*t*, 0) = 1 for *t* = 0, 1…, *n* and *I*(0, *p*) = 0 for *p* = 1, 2, …, *m*. We also set $\mathrm{vaf}(M_{0})=1$ and do not allow elimination of *M*_0_. Matrices *B* and *Y* are also expanded in an obvious way by allowing mutational indices to be equal to 0. The remainder of the tree topology is imposed through additional constraints that specify ancestor-descendant relationships in a consistent manner across all nodes.

1. We must satisfy the following constraints: (i) *K*(0) = 0, (ii) *Y*(*t*, 0) = 1 for *t* = 0, 1, …, *n*, and (iii) *Y*(0, *p*) = 0 for *p* = 1, 2, …, *m*.
2. If a mutation *p* is an ancestor of a mutation *q* and ISA holds for both *p* and *q*, then the true cellular prevalence of *p* is expected to be greater than or equal to the true cellular prevalence of *q*. Since vaf(*p*) and vaf(*q*) reflect cellular prevalences as discussed above, we expect that, in the implied evolutionary tree, vaf(p)(1+δ)≥vaf(q), where *δ* is some positive constant which allows for the noise typically present in the observed VAFs. In order to incorporate VAFs in our model, we introduce binary function *a*, such that *a*(*p*, *q*) = 1 only if *p* is an “ancestor” of *q*. By definition, we set *a*(*p*, *p*) = 0 for all *p* ∈ {0, 1, …, *m*}. The constraints that we need to introduce are thus as follows:
  - For any pair of distinct mutations *p*, *q*, we must satisfy the following two constraints to ensure that (i) only one of them could be the ancestor of the other, and (ii) if there is a cell in which they appear together, then one must be the ancestor of the other:
    (12) $$\begin{array}{c} a(\;p,q)+a(q,p)\leq \min\{1-K(p),1-K(q)\}\\ a(\;p,q)+a(q,p)\geq B(\;p,q,1,1)-K(p)-K(q) \end{array}$$
    (we remind the reader that, for any mutation *r*, *K*(*r*) = 1 indicates that the column *r* in input matrix *I* has been eliminated).
  - Each noneliminated mutation *q* different from null mutation must have at least one ancestor. This is ensured by adding the following constraint:
    (13) $$\overset{m}{\underset{\;p=0}{\sum }}a(\;p,q)\geq 1-K(q).$$
    On the other hand, a null mutation has no ancestors, so we set *a*(*p*, 0) = 0 for all *p* ∈ {0, 1, …, *m*}.
  - Consider two noneliminated mutations *p* and *q*. If *a*(*p*, *q*) = 1, then in genotype corrected output matrix *Y*, the column *p* should dominate the column *q*, that is, for each cell/row *r*, if the entry for *p* is 0, then the entry for *q* should also be 0. In other words, there should not exist row *r* such that *Y*(*r*, *p*) = 0 and *Y*(*r*, *q*) = 1, which is equivalent to *B*(*p*, *q*, 0, 1) = 0. To ensure this, for all pairs of mutation (*p*, *q*), we add the following constraint:
    (14) a(p,q)≤1−B(p,q,0,1).
  - If, for two noneliminated mutations *p* and *q*, matrix *Y* contains a cell in which *p* is present and *q* is absent (i.e., there exists *i* such that *Y*(*i*, *p*) = 1 and *Y*(*i*, *q*) = 0, which is equivalent to *B*(*p*, *q*, 1, 0) = 1), as well as a cell where both *p* and *q* are present (i.e., there exists *j* such that *Y*(*j*, *p*) = 1 and *Y*(*j*, *q*) = 1, which is equivalent to *B*(*p*, *q*, 1, 1) = 1), then *p* must be an ancestor of *q* (i.e., *a*(*p*, *q*) = 1). In order to ensure this, for all pairs of mutations (*p*, *q*), we add the following constraints:
    (15) a(p,q)≥B(p,q,1,0)+B(p,q,1,1)−1−K(p)−K(q).
  - For some small user-defined error tolerance value *δ* > 0 that accounts for variation in sequencing coverage, if vaf(q)>vaf(p)(1+δ) then *a*(*p*, *q*) = 0; in other words, for every pair of mutations *p* and *q* we must satisfy
    (16) $$\begin{array}{c} a(\;p,q)\cdot \mathrm{vaf}(p)\cdot (1+\delta )\geq a(\;p,q)\cdot \mathrm{vaf}(q). \end{array}$$
    If more than one sample from the same tumor with (independent) bulk sequencing data is available, we will have to satisfy the VAF constraints imposed by all of them. Let vaf_ℓ_(*p*) denote vaf(*p*) in sample ℓ. Then, for each pair of mutations *p* and *q* such that $\underset{\ell }{\mathrm{vaf}}(q)>\underset{\ell }{\mathrm{vaf}}(p)(1+\delta )$ we must satisfy: *a*(*p*, *q*) = 0; that is, for each sample ℓ:
    (17) $$\begin{array}{c} a(\;p,q)\cdot {\mathrm{vaf}}_{\ell }(p)\cdot (1+\delta )\geq a(\;p,q)\cdot {\mathrm{vaf}}_{\ell }(q) \end{array}$$
  - For all triplets of mutations *p*, *q*, *r*, we must ensure that, if *a*(*p*, *q*) = 1 and *a*(*q*, *r*) = 1, then *a*(*p*, *r*) = 1:
    (18) ∀p,q,r:a(p,r)≥a(p,q)+a(q,r)−1.
3. Now, we can describe our constraint for every triplet of distinct mutations *p*, *q*, and *r*, such that *p* is an ancestor of *q* and *r* but *q* and *r* do not have an ancestor-descendant relationship (i.e., *a*(*p*, *q*) = *a*(*p*, *r*) = 1 and *a*(*q*, *r*) = *a*(*r*, *q*) = 0).
  (19) vaf(p)⋅(1+δ)≥vaf(q)⋅[a(p,q)−a(r,q)−a(q,r)]+vaf(r)⋅[a(p,r)−a(r,q)−a(q,r)].
  If again, multiple samples with (independent) bulk sequencing data are available, we have to satisfy the triple-VAF constraint for each sample ℓ, that is, for each triplet of mutations *p*, *q*, *r*:
  (20) $${\mathrm{vaf}}_{\ell }(p)\cdot (1+\delta )\geq {\mathrm{vaf}}_{\ell }(q)\cdot [a(\;p,q)-a(r,q)-a(q,r)]+{\mathrm{vaf}}_{\ell }(r)\cdot [a(\;p,r)-a(r,q)-a(q,r)].$$

Note that the above *triple-VAF* constraint does not fully utilize the information provided by VAFs, for example, in case a *parent* mutation has three distinct *children* whose total VAFs should, in principle, not exceed that of the parent. It is possible to generalize the triple-VAF constraint to any number of children (see Supplemental Material). Nevertheless, we still recommend the use of the triple-VAF constraint instead of this general-VAF constraint (even though this choice may, in principle, produce trees that violate the general-VAF constraint) since the two sets of constraints do not seem to produce different trees in practice. Furthermore, the general-VAF constraint is quadratic and thus slows down PhISCS.

### PhISCS-B for tumor phylogeny inference via SCS data

We first show how to reduce the ILP formulation of PhISCS where only single-cell data is used as the input and no mutation elimination allowed to a wMax-SAT problem. For each input entry *I*(*i*, *j*), 1≤i≤n,1≤j≤m, we introduce a Boolean variable *Y*(*i*, *j*) which represents the true state of mutation *j* in cell *i*. Our goal is to find the assignment of values to variables *Y*(*i*, *j*) such that the resulting matrix *Y* is conflict-free and the objective defined below is maximized. In order to enforce that *Y* is conflict-free matrix, we use a set of additional Boolean variables *B*(*p*, *q*, *a*, *b*) (analogous to binary variables used in earlier sections) that need to satisfy the following *hard* constraints (the constraints that *need* to be satisfied):

(21) $$\begin{array}{rl} & \neg (Y(i,p)\wedge Y(i,q)\wedge \neg B(\;p,q,1,1))\\ & \neg (\neg Y(i,p)\wedge Y(i,q)\wedge \neg B(\;p,q,0,1))\\ & \neg (Y(i,p)\wedge \neg Y(i,q)\wedge \neg B(\;p,q,1,0))\\ & \neg (B(\;p,q,0,1)\wedge B(\;p,q,1,0)\wedge B(\;p,q,1,1)). \end{array}$$

We can now define our objective as satisfying all the hard constraints with alterations on the input matrix *I* with maximum probability, where each alteration (indicating a false positive or false negative) is independent. This objective corresponds to the minimizing the (weighted) number of flipped entries in the solution matrix *Y* in comparison to *I*, or, for the purpose of formulating the problem as an instance of wMax-SAT, maximizing the weighted sum of the following “soft” constraints (for all *i*, *j*, s.t. *I*(*i*, *j*) ≠ ? originally):

(22) $$\begin{array}{rl} \mathrm{if}\;\;\;I(i,j) & =0\;\mathrm{weight}\;\;\;\mathrm{for}\;\;\;Y(i,j)\;\mathrm{is}:\;\log\frac{\text{β}}{1-\alpha }\\ \mathrm{if}\;\;\;I(i,j) & =1\;\mathrm{weight}\;\;\;\mathrm{for}\;\;\;Y(i,j)\;\mathrm{is}:\;\log\frac{1-\text{β}}{\alpha } \end{array}.$$

Note that, in order to get exactly the same objective value as in objective defined in Equation 4, we need to add constant terms from Equation 4 to the objective defined in Equation 22. Alternatively, after solving for matrix *Y*, we can compute *P*(*I*|*Y*) by the use of formula given in Equation 2.

We now show how to account for ISA violations: For each column *j* ∈ {1, 2, …, *m*} we introduce a Boolean variable *K*(*j*) that is set to 1 if and only if column *j* is eliminated (i.e., mutation corresponding to column *j* is not considered as a part of the output).

Analogously as in the ILP formulation, we allow at most *k*_max_ columns to be eliminated, where *k*_max_ is a user-defined constant. In order to ensure that no more than *k*_max_ of variables *K*(1), *K*(2), …, *K*(*m*) are set to 1, for each possible (*k*_max_ + 1)-tuple $\left(i_{1},i_{2},\ldots ,i_{k_{\max\;}+1}\right)$ of integers such that $1\leq i_{1}<i_{2}<\ldots <i_{k_{\max\;}+1}\leq m$, we add the following hard clause

(23) $$\neg (K(i_{1})\wedge K(i_{2})\wedge \ldots \wedge K(i_{k_{\max}+1}))$$

to our model.

Now, for any eliminated column *p*, we do not have to check whether it is in conflict with any other column *q* or vice versa. Therefore, for each pair (*p*, *q*) of columns, we replace the last constraint in 21 above with the following:

(24) ¬(¬K(p)∧¬K(q)∧B(p,q,0,1)∧B(p,q,1,0)∧B(p,q,1,1)).

To get the objective equivalent to Equation 10, for each pair of cell *i* and mutation *j*, we introduce a binary variable *X*(*i*, *j*) and add the following hard constraint:

(25) $$\begin{array}{r} (\neg Y(i,j)\vee \neg K(j)\vee X(i,j))\wedge (Y(i,j)\vee \neg X(i,j))\wedge (K(j)\vee \neg X(i,j) \end{array})$$

and transform Equation 10 to an instance of wMax-SAT where the goal is to maximize weighted sum of the following “soft” constraints (for all *i*, *j*, s.t. *I*(*i*, *j*) ≠ ?):

(26) $$\begin{array}{rl} \mathrm{if}\;\;\;I(i,j) & =0\;\mathrm{weight}\;\;\;\mathrm{for}\;\;\;\neg K(j)\;\mathrm{is}:\;\;\;\;\;\log(1-\alpha )\\ \mathrm{if}\;\;\;I(i,j) & =1\;\mathrm{weight}\;\;\;\mathrm{for}\;\neg K(j)\;\;\;\mathrm{is}:\;\;\;\;\log(\alpha )\\ \mathrm{if}\;\;\;I(i,j) & =0\;\mathrm{weight}\;\;\;\mathrm{for}\;\;\;X(i,j)\;\mathrm{is}:\;-\log\frac{\text{β}}{1-\alpha }\\ \mathrm{if}\;\;\;I(i,j) & =1\;\mathrm{weight}\;\;\;\mathrm{for}\;X(i,j)\;\;\;\mathrm{is}:\;-\log\frac{1-\text{β}}{\alpha }\\ \mathrm{if}\;\;\;I(i,j) & =0\;\mathrm{weight}\;\;\;\mathrm{for}\;\;\;Y(i,j)\;\mathrm{is}:\;\;\;\log\frac{\text{β}}{1-\alpha }\\ \mathrm{if}\;\;\;I(i,j) & =1\;\mathrm{weight}\;\;\;\mathrm{for}\;Y(i,j)\;\;\;\mathrm{is}:\;\;\;\log\frac{1-\text{β}}{\alpha }. \end{array}$$

### Additional Boolean constraints to integrate VAFs derived from bulk sequencing data into PhISCS-B

In order to integrate information derived from bulk sequencing data, represented in the form of VAFs of the given set of mutations, we explicitly impose a tree structure on the output matrix *Y* through the use of a number of Boolean constraints.

The Boolean constraints below start by defining the *root node* via a new row, indexed by 0, that represents the genotype of a normal cell. We also add a new column, indexed by 0, and associated *null mutation M*_0_ which represents a mutation specific to the normal cell or, in other words, a germline SNP present in all cells. We set *I*(*t*, 0) = 1 for *t* = 0, 1…, *n* and *I*(0, *p*) = 0 for *p* = 1, 2, …, *m*. We also set $\mathrm{vaf}(M_{0})=1$ and do not allow elimination of *M*_0_. The remainder of the tree topology is imposed through additional constraints that specify ancestor-descendent relationships in a consistent manner across all nodes:

1. We must satisfy the following constraints which can easily be converted into Boolean expressions: (i) *K*(0) = 0, (ii) *Y*(*t*, 0) = 1 for *t* = 0, 1, …, *n*, and (iii) *Y*(0, *p*) = 0 for *p* = 1, 2, …, *m*.
2. If a mutation *p* is an ancestor of mutation *q* in the implied evolutionary tree, then vaf(p)≥vaf(q) within some relatively small error tolerance. In order to employ VAFs using the above constraints between mutational pairs, we introduce Boolean function *a* such that *a*(*p*, *q*) = 1 if and only if *p* is an ancestor of *q*. The hard constraints that need to be imposed on *a* are as follows:
  a. For all pairs of distinct mutations *p* and *q*, where both *p* and *q* are different from null mutation, we must satisfy
    (27) $$\begin{array}{r} \begin{array}{c} a(\;p,q)\vee a(q,p)\Rightarrow \neg K(p)\wedge \neg K(q)\\ \neg [\left(a(\;p,q)\wedge a(q,p)\right)]\\ B(\;p,q,1,1)\Rightarrow a(\;p,q)\vee a(q,p). \end{array} \end{array}$$
  b. For each noneliminated mutation *q* different from null mutation, we must make sure that it has an ancestor mutation (which could be null mutation). This is achieved by imposing the following constraint:
    (28) $$\left(\underset{\forall p\neq q}{\vee }a(\;p,q)\right)\vee K(q).$$
  c. Consider two noneliminated mutations *p* and *q*. If *a*(*p*, *q*) = 1, then in genotype corrected output matrix *Y*, the column *p* should dominate the column *q*, that is, for each cell/row *r*, if the entry for *p* is 0 then the entry for *q* should also be 0. In other words, there should not exist row *r* such that *Y*(*r*, *p*) = 0 and *Y*(*r*, *q*) = 1, which is equivalent to *B*(*p*, *q*, 0, 1) = 0. To ensure this, for all pairs of mutation (*p*, *q*), we add the following constraint:
    (29) ¬a(p,q)∨¬B(p,q,0,1)∨K(p)∨K(q).
  d. If, for two noneliminated mutations *p* and *q*, matrix *Y* contains cells in which *p* is present and *q* is absent (i.e., there exists *i* such that *Y*(*i*, *p*) = 1 and *Y*(*i*, *q*) = 0, which is equivalent to *B*(*p*, *q*, 1, 0) = 1), as well as cells where both *p* and *q* are present (i.e., there exists *j* such that *Y*(*j*, *p*) = 1 and *Y*(*j*, *q*) = 1, which is equivalent to *B*(*p*, *q*, 1, 1) = 1), then *p* must be the ancestor of *q* (i.e., *a*(*p*, *q*) = 1). In order to ensure this, for all pairs of mutations (*p*, *q*), we add the following constraints:
    (30) (B(p,q,1,0)∧B(p,q,1,1))⇒(a(p,q)∨K(p)∨K(q)).
  e. For some small user-defined error tolerance value *δ* > 0 that accounts for variation in bulk sequencing coverage, if vaf(q)>vaf(p)⋅(1+δ), then *a*(*p*, *q*) = 0; in other words, for each pair of mutations *p* and *q* for which *a*(*p*, *q*) = 1, we must satisfy vaf(p)⋅(1+δ)≥vaf(q). In order to express this as a Boolean constraint, we introduce a new Boolean function Pvaf(p,q) defined for all pairs of mutations *p* and *q* (as a part of the input specification) as follows:
    (31) Pvaf(p,q)=1, if vaf(p)⋅(1+δ)≥vaf(q)=0, otherwise.
    Then, the constraint that must be satisfied for each pair of mutations *p* and *q* is
    (32) $$\begin{array}{c} a(\;p,q)\Rightarrow \mathrm{Pvaf}(p,q). \end{array}$$
    If more than one sample from the same tumor with (independent) bulk sequencing data is available, we will have to satisfy the VAF constraints imposed by all of them. Specifically, let vaf_ℓ_(*p*) denote vaf(*p*) in sample ℓ. Then, for each pair of mutations *p* and *q*, Pvaf(p,q)=1 only if $\underset{\ell }{\mathrm{vaf}}(p)\cdot (1+\delta )\geq \underset{\ell }{\mathrm{vaf}}(q)$ for all samples ℓ, and Pvaf(p,q)=0, otherwise.
  f. For all triplets of mutations *p*, *q*, *r*, we must ensure that, if *a*(*p*, *q*) = 1 and *a*(*q*, *r*) = 1, then *a*(*p*, *r*) = 1
    (33) ∀p,q,r:a(p,q)∧a(q,r)⇒a(p,r).
3. For all triplets of distinct mutations *p*, *q*, and *r* such that *p* is an ancestor of *q* and *r* but *q* and *r* do not have an ancestor-descendent relationship (i.e., they belong to different lineages in the tree), we must satisfy vaf(p)⋅(1+δ)≥vaf(q)+vaf(r). In order to express this as a Boolean constraint, we introduce yet another Boolean function Tvaf(*p*, *q*, *r*) defined for all triplets of mutations *p*, *q*, *r* (as a part of the input specification) as follows:
  (34) Tvaf(p,q,r)=1, if vaf(p)⋅(1+δ)≥vaf(q)+vaf(r)=0, otherwise.
  Then, the constraint that must be satisfied for all mutations *p*, *q*, *r* is
  (35) [a(p,q)∧a(p,r)∧¬a(q,r)∧¬a(r,q)]⇒Tvaf(p,q,r).
  If multiple samples from the same tumor with (independent) bulk sequencing data are available, we will have Tvaf(p,q,r)=1 if $\underset{\ell }{\mathrm{vaf}}(p)\cdot (1+\delta )\geq \underset{\ell }{\mathrm{vaf}}(q)+\underset{\ell }{\mathrm{vaf}}(r)$ for all ℓ.

### Software availability

PhISCS is available at https://github.com/sfu-compbio/PhISCS and as Supplemental Code.

## Supplementary Material

Supplemental Material
