# Supplementary material for: PhISCS: a combinatorial approach for subperfect tumor phylogeny reconstruction via integrative use of single-cell and bulk sequencing data
Source: Genome Res. 2019 Nov;29(11):1860–77. doi: 10.1101/gr.234435.118 (PMC6836735; doi:10.1101/gr.234435.118)
Supplement: Supplemental Material [file supp_29_11_1860__index.html]

PhISCS: a combinatorial approach for subperfect tumor phylogeny reconstruction via integrative use of single-cell and bulk sequencing data — PhISCS: a combinatorial approach for subperfect tumor phylogeny reconstruction via integrative use of single-cell and bulk sequencing data — Supplemental Material 

# PhISCS: a combinatorial approach for subperfect tumor phylogeny reconstruction via integrative use of single-cell and bulk sequencing data

## Supplemental Material

- Supplemental\_Material.pdf
- Supplemental\_Code.zip
