# Supplementary material for: PhISCS: a combinatorial approach for subperfect tumor phylogeny reconstruction via integrative use of single-cell and bulk sequencing data
Source: Genome Res. 2019 Nov;29(11):1860–77. doi: 10.1101/gr.234435.118 (PMC6836735; doi:10.1101/gr.234435.118)
Supplement: Supplemental Material [file supp_gr.234435.118_Supplemental_Material.pdf]

Supplemental Material

PhISCS: A Combinatorial Approach for Sub-perfect Tumor Phylogeny Reconstruction via Integrative use of Single-cell and Bulk Sequencing Data

Malikic et al.

A Computational Estimate for the (Maximum) Number of ISA Violating Mutations, k_{max}

PhISCS asks the user to specify k_{max}, the maximum number of ISA violating mutations it can tolerate. If k_{max} is not known in advance, it is possible to estimate it through the following heuristic that employs PhISCS - with no ISA violations allowed. Given the tumor phylogeny T obtained with no ISA violations, for each mutation m, compute l, the number of *descendant* mutations of m in T, i.e. the number of mutations m' which only appear in a subset of cells that harbor m in the inferred mutation matrix Y. Now let k' be the number of cells in Y harboring m and let k be the number of cells in the input matrix I which do not harbor m but harbor at least one of the l descendants of m. Consider the probability of k or more cells out of k' descendants of m to have "lost" m as

$$p = \sum_{i=k}^{i=k'} \beta^i (1-\beta)^{k'-i}$$

where β is the false negative rate. If k deviates from $Exp(p)$ (the expected value of p) by at least three times the standard deviation (or any other user defined value), it can be identified as a potential ISA violator. The total number of potential ISA violators can then be used as an estimate on the number of ISA violations allowed. Note that even with a user defined value of k_{max}, it is possible to use the above approach to constrain the mutations that can be considered as an ISA violator to those identified as potential ISA violators. When applied to simulated data sets, PhISCS equipped with this additional constraint better identifies ISA violating mutations.

Generalizing the Triple-VAF Constraints to Arbitrary Number of Mutations

It is possible to generalize the VAF constraint on mutation triplets to larger sets by defining a new function $pa(p,q)$ which would be set to 1 if p is the parent of q and 0 otherwise as follows:

$$\begin{array}{c} \forall p,q:\ a(p,q) \geq pa(p,q) \\ \forall p,q:\ 1-pa(p,q) \geq \sum_{\forall r}[a(r,p)(1-a(r,q)) + a(r,q)(1-a(r,p))] \\ \forall q \neq 0:\ \sum_{\forall p} pa(p,q) = 1 \end{array} \tag{1}$$

Now we introduce the constraint for the children of a mutation p:

$$\forall p:\ \text{vaf}(p)\cdot(1+\delta) \geq [\sum_{\forall q} pa(p,q)\cdot \text{vaf}(q)] \tag{2}$$

Simulation Models used for Benchmarking Tumor Phylogeny Inference Methods

We can simulate the history of tumor progression and subclonal composition through the use of the following (the notation is borrowed from (Malikic et al. 2019) and for simplicity we will first assume that single bulk sample is simulated):

- Rooted phylogenetic tree T with the set of nodes $S(T)$ of size s, where the root node represents the population of healthy cells and each of the remaining nodes represents a distinct population of tumor cells (i.e. a subclone) emerging through selective sweeps during the course of tumor evolution. This tree can be represented by the ancestry matrix A_T defined as follows:

$$\begin{aligned} A_T[i,j] &= 1, \text{ if node } i \text{ is an ancestor of node } j \text{ or } i=j \\ &= 0, \text{ otherwise.} \end{aligned}$$

- Set $M = \{M_1, M_2, \ldots, M_m\}$ representing mutations that occur during the course of tumor evolution.

- Function $N_T : M \to S(T)$ where $N_T(x)$ denotes the node (i.e. subclone) in tree T where mutation x occurs for the first time.

- Set $F = \{f_1, f_2, \ldots, f_s\}$ where, for each $i \in \{1,2,\ldots,s\}$, f_i is a non-negative real number that represents the frequency of the cellular population corresponding to node i of tree T. Obviously, frequencies f_i must satisfy $\sum_{i=1}^{s} f_i = 1$.

In our simulations, we restrict ourselves to heterozygous single-nucleotide variants (SNVs) from diploid regions of the genome.

Under the ISA, for a mutation M_i we define its cellular prevalence $h(M_i)$ as a sum of frequencies of cellular populations harboring M_i. According to our notation, $h(M_i)$ can be expressed as

$$h(M_i) = \sum_{j=1}^{s} A_T[N_T(M_i), j] \cdot f_j.$$

Finally, for node $t \in T$ we define its genotype, denoted as G_t, as a row binary vector of length m such that $G_t[i] = 1$ only if M_i is present in the subclone corresponding to node t, which is equivalent to mutation M_i emerging at the node on the path between the root and node t (inclusively). More formally, $G_t[i] = A_T[N_T(M_i), t]$.

Based on the above notation, and using model of clonal tumor evolution and bulk-data simulation presented in (Malikic et al. 2015) and (Malikic et al. 2019), we generate tree T of size s by randomly choosing one of the possible rooted tree topologies of size s (that were made available in (Malikic et al. 2015)). We then choose values of f_i by formula

$$f_i = 0.05 + (1 - 0.05 \cdot s) \cdot \frac{x_i}{\sum_{i=1}^{s} x_i}$$

where x_i for $i \in \{1,2,\ldots,s\}$ are randomly chosen real numbers from the interval $(0,1)$. The above constant 0.05 ensures the minimal subclonal frequency of 5%. Finally, we randomly spread 40 mutations across the nodes of T, excluding root, and such that each node gets assigned at least one mutation in order to avoid nodes with identical genotypes. We repeat the above simulations 10 times for each $s \in \{4, 7, 10\}$.

For mutation M_i we simulate bulk-sequencing read counts from binomial distribution with parameters t (number of trials) and $\frac{h(M_i)}{2}$ (success probability), where t represents sequencing coverage. Value of $h(M_i)$ is divided by 2 in the success probability parameter of binomial distribution due to the assumption that M_i is heterozygous SNV from diploid region of the genome.

For a given tree of tumor evolution and subclonal frequencies simulated above, SCS data is simulated by first drawing 100 single cells from $s-1$ cancerous populations proportional to their subclonal frequencies and using subclone mutational profiles encoded by row vectors G_t, where $t \in S(T)$. After that, we first simulate missing(non-observed) entries of SCS data matrix with rate of 0.10. Next, we simulate false positive entries using the false positive rate of 0.0001. Finally, we introduce false negative sequencing noise by using false negative rates of 0.15 and 0.25 each. This is repeated for all 30 simulations generated above resulting in 60 different simulations.

In order to simulate violations of ISA, for each of the 60 simulations described above, we first generate simulations where exactly one of the mutations violates ISA. In order to do this, we randomly choose mutation M_i and assign it to node p different from root and $N_T(M_i)$. Depending on the relation between p and $N_T(M_i)$ this violation might represent recurrence or loss of previously obtained mutation. In either case we update genotype of each node and value $h(M_i)$ (in an obvious way) and repeat the above procedure of generating bulk and SCS data.

For each of 60 simulations from the previous step, each having exactly one mutation M_i violating ISA, we choose another mutation $M_j \neq M_i$ from set M and apply the same procedure as above simulating two mutations violating ISA (in our simulations ISA can be violated due to parallel mutation, loss of heterozygosity or deletion).

180 simulations generated in the previous steps are later used as the input for benchmarking.

When simulating multiple bulk samples, since all cancerous cells are assumed to originate from single cancer founder cell, we assume that all samples share the same phylogeny (see (Malikic et al. 2015)) and generate subclonal frequencies f independently for each sample using the above procedure. Based on these frequencies in a given sample, read counts for that particular sample are then drawn analogously as described above. Weight of each subclone used when drawing single cells is obtained by averaging its frequencies over all bulk samples.

Simulations involving Copy Number Altered Mutations

The simulations above assume that no copy number alterations (detectable or undetectable) would impact the genes involved in our analysis. In real data analysis however, even after a pre-processing step to profile CNAs followed by a filtering of mutations from non-diploid regions, CNA impacted (especially subclonal) mutations may remain in data. Undetected CNAs are particularly likely in the cases where CNA of small scale (gain of 1 or 2 copies) affects small genomic region not containing germline SNPs (which are frequently used for inferring allelic imbalance and changes in copy number). For this reason, in our simulation we allow only gain of 1 or 2 copies of genomic region. We also assume that copy number gains precede the SNVs occurrence and, based on the number of copies gained, adjust success probability for variant reads. Note that the loss of heterozygosity or loss of mutation due to deletion of genomic region harboring it are already modelled in our simulations via the violations of ISA.

In order to assess the performance of PhISCS under such CNAs, we simulated data with 40 somatic SNVs with varying number of bulk samples (1, 3 and 5). The number of subclones and single cells were set to 7 and 100, respectively. We varied the false negative rates of SCS data, while the bulk data coverage was set to $5,000\times$. In each case, we assumed that ISA is violated for 1 or 3 mutations. In addition to simulating violations of ISA, we also assumed that a small subset of SNVs (3 out of 40), falls into genomic regions affected by clonal copy number gains.

TPTED **measure for comparing tumor phylogenies**

Given two tumor phylogenies T and G, where T is an inferred mutation tree (thus every node has a single, unique label) and G is a ground truth tree (thus some nodes may have more than one label but each label occurs once), we define $\text{TPTED}(T,G) = \text{TED}(T,G')$ where $G' = F(G)$ and $F = \arg\min_f \text{TED}(T, f(G))$ among all functions f that transform the multi-labeled tree G to a mutation tree G' in a way that each node with k labels is replaced by a path of singly labeled nodes of length k. Note that TED between trees T' and G' where each node is singly labeled, is the minimum number of node/label deletions on T' and G'. Here the deletion of a node results in its children becoming the children of its parent - thus the deletion of the root is not permitted. Without loss of generality we can assume that the roots of T and G have the same label representing the germ line. For any such function f, we have by definition $\text{TED}(T, f(G)) \geq \text{TPTED}(T,G)$.

Below we show that a function F which minimizes $\text{TED}(T, f(G)$ is efficiently computable, reducing the problem of computing TPTED between T and G to that of computing TED - which can easily be done through dynamic programming (as mentioned earlier, TED is NP-hard to compute in general; however in our application each label appears once in each tree).

F is computed as follows. For each node v in G with labels $L = \{l_1, l_2, \ldots, l_x\}$, consider the corresponding sequence of nodes $\{w_1, w_2, \ldots, w_x\}$ in T, ordered with respect to breadth first search traversal of T. F partitions v into a path of exactly x nodes $v_1, v_2, \ldots, v_x$, with respective labels $l_1, l_2, \ldots, l_x$ and v_1 being closest to the root. Consider the mutation tree $G' = F(G)$ as defined above. Then, as will be shown below, $\text{TPTED}(T,G) = \text{TED}(T,G')$.

Theorem 1. *The function F above satisfies that for any mutation tree T and any other tree G with the same label set where each label appears once but each node may have more than one label,* $\text{TED}(T, F(G)) = \text{TPTED}(T,G)$.

Proof. Assume, without loss of generality, that T has n nodes labeled by $\{1,2,\ldots,n\}$ such that the ordering of labels of T with respect to its breath first traversal (BFS) is $1,2,\ldots,n$. Consider the "transformation" function F_T on G which is a sequence of transformations $\{f_1, f_2, \ldots f_n\}$ on G such that each f_i partitions the node v of G that includes label i ($1 \leq i \leq n$) into two, provided that v includes at least two labels, such that the new *parent node* v' is assigned label i and the *child node* v'' is assigned the remaining of the labels of v - f_i does not alter G if i is the only label of node v. Now we show by induction that $\text{TED}(T, F_T(G)) = \text{TED}(T, F(G))$, i.e. has the minimum possible value.

For the base case, we assume that T and G each have one vertex with label 1. Necessarily $F_T(G) = G$ and $\text{TED}(T, f(G)) = \text{TPTED}(T,G) = 0$. For the inductive step, suppose that our claim is true for any pair of trees g and t with $l-1$ labels i.e. $\text{TED}(t, F_t(g)) = \text{TPTED}(t,g)$. Given trees T and G with l labels each, there must exist trees t and g with $l-1$ labels such that T extends t by adding to it a single vertex w that is the last in the BFS ordering of its vertices - with label l, and G extends g by adding label l to its vertex v. Consider the greatest valued label i in vertex

v. In case $i = l$, F_T on G extends F_t on g by adding one final transformation f_l which does not alter G. Otherwise F_T extends F_t by altering f_i so that it now splits node v in two such that the new parent v' is assigned label i and the new child v'' is assigned label l - since l is the very last label to be encountered in the BFS of T. Denote $F_T(G)$ by G' and assume that, as a result of f_i, $\text{TED}(T, G') > \text{TPTED}(T, G)$. This implies by our inductive assumption that $\text{TED}(T, G') = \text{TED}(T, G'') + 1 = \text{TPTED}(T, G) + 1$ where G'' is identical to G' with the single exception that in G'', the child node v'' is assigned label i and the parent node v' is assigned label l rather than the converse. If this is the case, there must exist a label j that lies in the path between the node of l and the node of i in T, which is assigned to an ancestor of v in G. However since TED between a linear tree topology Θ with three nodes and respective labels i, j, l, and an identical tree topology Γ with respective labels j, i, l, is equal to $\text{TED}(\Theta, \Gamma')$ where Γ' is again a linear topology with respective labels j, l, i (they are both $= 1$), it must be the case that $\text{TED}(T, G') = \text{TED}(T, G'')$. $\square$

Benchmarking SCITE, SiFit, B-SCITE and PhISCS

SCITE and SiFit were both run using default parameter settings, with the exception of chain length in SCITE. In particular, for SCITE we set the number of repetitions to 1 and the chain length of MCMC repetitions to 900,000 (which is 10 times higher than default value and is done in order to allow better convergence). As SiFit models violations of ISA and the number of cells used in our simulations is typically larger than the number of simulated mutations, the search space that SiFit considers might be considerably larger. For these reasons, in all of the experiments, when running this tool we allowed 3 repeats, each with 10,000,000 iterations.

In order to mimic real settings, where only approximate estimates of false negative (FN) and fale positive (FP) error rates are available, in each case we first added noise to true FN and FP error rates and provide resulting noisy values of these parameters as input to each of PhISCS, SCITE and SiFit. The noise was added as follows: if FN error rate used to generate simulations equals β, the noisy value used as parameter was $\beta \times \mathcal{N}(1, 0.1)$ where $\mathcal{N}(1, 0.1)$ is a random number derived from a normal distribution with mean 1, standard deviation 0.1 and from the interval $(0.5, 2)$ (draws from Normal distribution are repeated in the cases where number falls outside of this range). Analogous was done for adding noise to FP error rates.

For the simulation depicted in Figures 1, 2, 3, 4, 5 and 6 (when ISA violations are allowed and both single-cell and bulk data were available) we respectively set δ to 0.01, 0.05, 0.05, 0.20, 0.01 and 0.20.

For the simulation depicted in Figure 6, we allowed B-SCITE to run for 24 hours for each run. The other parameters were `-dr 0 -e 0.20 -w 0.50 -abr 0.50`.

In all computations of MLTD and MLTSM measures, from each of the two input trees that we compare (i.e. true and inferred tree), we removed mutations eliminated in the inferred tree, as well as mutations that violate ISA in the true tree. This step allows fairer comparison and is particularly advantageous for SCITE and B-SCITE. Otherwise, regardless of the tree that these two methods report, MLTD measure would be at least equal to the number of ISA violating mutations in the true tree.

Details related to obtaining and pre-processing "real" data

The single-cell data matrix for CRC2 was made available in the Supplementary Figure 7 of the original study (Leung et al. 2017). All single cells without any detected mutation were filtered from the input, as they are non-informative for PhISCS.

In order to obtain bulk data read counts (which we could not find in the original study), we first downloaded primary and metastatic aneuploid whole exome sequencing samples for each patient (raw data available at SRA, runs: SRR3472569, SRR3472571, SRR3472800 and SRR3472796). This was followed by read alignment (using Bowtie 2 (Langmead and Salzberg 2012)), duplicates removal (using Picard tools) and filtering of reads with mapping quality lower than 40. Since for each mutation of interest, reference and variant nucleotide for its genomic position were provided in (Leung et al. 2017), number of reads supporting variant and reference alleles could be obtained directly from the read alignment files. Mutations having less than 20 reads in total in each of the primary and metastasis sample were excluded from the analysis as their VAFs are severly affected by variance (e.g. at coverage $20\times$ only a single variant read difference creates VAF difference of 0.05). Note that the filtering described here is the same as was previously used in (Malikic et al. 2019). In the next section we also present results of the analysis obtained by PhISCS when no such filtering of mutations is applied.

For ALL patient, both single-cell data matrix and bulk data VAFs were made available in the original study. In order to filter doublets (that were previously reported to be present in this dataset (Kuipers et al. 2017)), before running PhISCS, we first run Single Cell Genotyper (Roth et al. 2016). This resulted in filtering of 13 cells from the original input (that consists of 115 single cells).

A re-analysis of the CRC2 patient data including low coverage mutations

In the main manuscript we summarize results obtained by PhISCS on the SCS data set previously used in (Malikic et al. 2019); on this data B-SCITE, which does not allow ISA violations, could not find a tree topology that jointly explains all aspects of the single-cell and bulk data, but PhISCS can. Even though bulk sequencing data is also available for this tumor sample, because of the largely non-diploid nature of the genome of this tumor, VAFs of mutations (computed from whole exome sequencing data) were not directly used but rather were employed in a post-processing step to assess the predicted pairwise ordering of mutations.

We have also performed an expanded analysis that includes all mutations reported by (Leung et al. 2017), i.e. without filtering those mutations with low bulk sequencing covereage. Note that the placement of trunk mutations and mutations forming independent lineages close to the root of the tree, as shown in the Figure 6 of the original study (Leung et al. 2017), is not debatable[1], we focused our analysis on the remaining set of mutations.

On this set of mutations, PhISCS, under various values of false positive and false negative noise rate parameters, eliminates that in gene *ATP7B* (see Supplemental Figure 1). For each of the sites, the total number of false positives across all cells, as reported by PhISCS was usually 0 or 1, as can be expected from a typical SCS experiment (e.g. diploid cells sequenced in this study exhibited only a few false positives). Additionally, even though an elevated false negative rate is reported for a few of the mutated sites, it was still within the range that can be observed in single-cell data (the overall false negative rate reported by PhISCS is $\sim 15\%$). Additionally, the solution by PhISCS does not present any obvious contradictions in the ordering of mutations with respect to their VAFs derived from bulk data.

In summary, the topology inferred when considering the above set of mutations appears highly similar to the topology presented in the Figure 7 and none of the main conclusions that we made when discussing Figure 7 is changed. As for the placement of mutation *ATP7B*, PhISCS currently does not provide a placement for eliminated mutations. However, it may be possible to place an eliminated mutation by a post-processing of the reported solution. For this solution for example, based on the genotypes of single cells, our estimate is that *ATP7B* is gained in the primary site but affected by loss at some point in time in the metastatic site (and this is further supported by the observed VAF of this mutation).

[1]The mutations that we refer to as trunk mutations in that figure are all mutations, starting from the mutation in gene *TP53* down to the mutation in gene *MN1*, whereas the independent lineages are formed by the mutations in genes *ALK*, *ATR*, *EPHB6*, *NR3C2*, *SPEN* and *CIITA*.

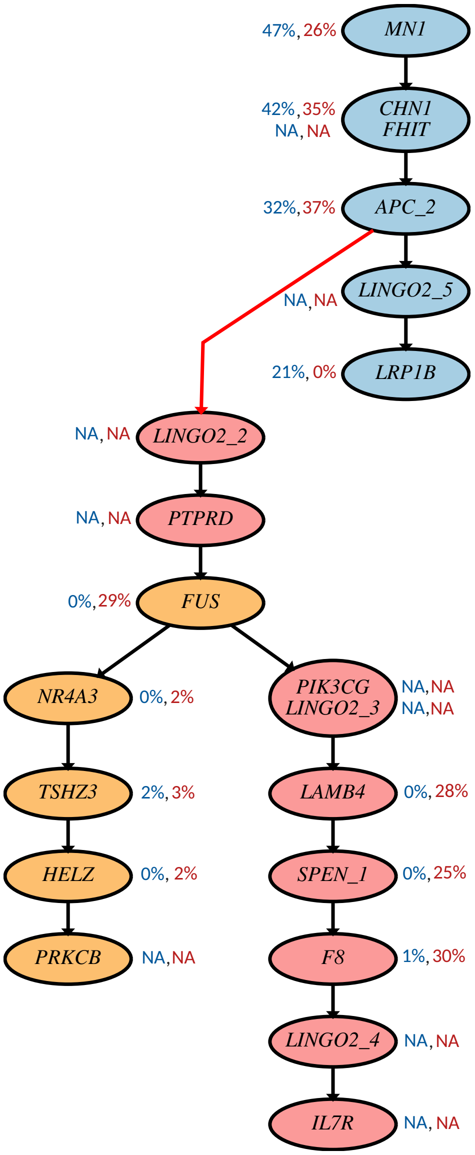

Supplemental Figure S1. Results of the analysis obtained by PhISCS for CRC2 patient from (Leung et al. 2017). We focused on the analysis of evolutionary dependencies between mutations involved in metastatic seeding, as the placement of the other mutations (i.e., trunk mutations and mutations from the independent lineage close to the root, see Figure 6 in (Leung et al. 2017)), is not debatable and is strongly supported by the data. For that reason, from trunk mutations and mutations forming the independent lineage, we only show the (trunk) mutation in gene *MN1*. In this solution, PhISCS again eliminated mutation in gene *ATP7B* and the key findings discussed in our main paper apply to this solution. Variant allele frequencies of mutations (having coverage not lower than 20× in at least one of the primary or metastatic bulk samples) can be found in the Figure 7. Genes *SPEN*, *APC* and *LINGO2* harbor multiple mutations. *SPEN_1* denotes mutation at position (Chr 1, 16258997), *APC_2* mutation at (Chr 5, 112175328), *LINGO2_2* mutation at (Chr 9, 29123273), *LINGO2_3* mutation at (Chr 9, 28778389), *LINGO2_4* mutation at (Chr 9, 28064983) and *LINGO2_5* mutation at (Chr 9, 29178757). Coloring of nodes is motivated by the coloring used in Figure 6 in (Leung et al. 2017). In this coloring, blue nodes represent mutations specific to the primary site, whereas nodes colored in pink and orange represent mutations specific to two distinct metastases as reported in (Leung et al. 2017). Red edge represents metastatic seeding event.

Source codes of Max-SAT solvers used for the implementation of CSP formulation of PhISCS

Source codes of Max-SAT solvers used for the implementation of CSP formulation of PhISCS (i.e. PhISCS-B) are available at:

- Z3: `https://github.com/Z3Prover/z3`
- MaxHS and MAXINO: `http://mse17.cs.helsinki.fi/descriptions.html`

References

Kuipers J, Jahn K, Raphael BJ, and Beerenwinkel N 2017. Single-cell sequencing data reveal widespread recurrence and loss of mutational hits in the life histories of tumors. *Genome research*, .

Langmead B and Salzberg SL 2012. Fast gapped-read alignment with bowtie 2. *Nature methods*, **9**(4):357.

Leung ML, Davis A, Gao R, Casasent A, Wang Y, Sei E, Vilar E, Maru D, Kopetz S, and Navin NE, *et al.* 2017. Single-cell dna sequencing reveals a late-dissemination model in metastatic colorectal cancer. *Genome research*, **27**(8):1287–1299.

Malikic S, Jahn K, Kuipers J, Sahinalp SC, and Beerenwinkel N 2019. Integrative inference of subclonal tumour evolution from single-cell and bulk sequencing data. *Nature communications*, **10**(1):2750.

Malikic S, McPherson AW, Donmez N, and Sahinalp SC 2015. Clonality inference in multiple tumor samples using phylogeny. *Bioinformatics*, **31**(9):1349–1356.

Roth A, McPherson A, Laks E, Biele J, Yap D, Wan A, Smith MA, Nielsen CB, McAlpine JN, Aparicio S, *et al.* 2016. Clonal genotype and population structure inference from single-cell tumor sequencing. *Nat Meth*, **13**(7):573–576. Brief Communication.
