# Supplementary figures and images for: PhISCS: a combinatorial approach for subperfect tumor phylogeny reconstruction via integrative use of single-cell and bulk sequencing data
Source: Genome Res. 2019 Nov;29(11):1860–77. doi: 10.1101/gr.234435.118 (PMC6836735; doi:10.1101/gr.234435.118)

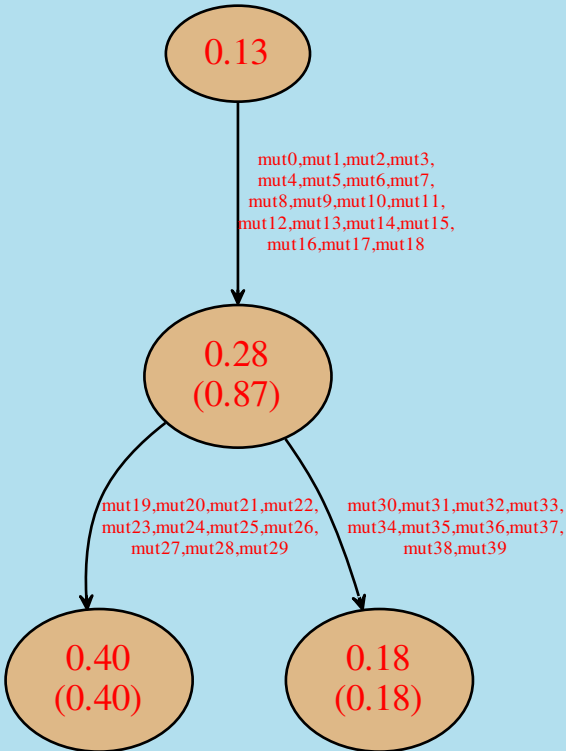

Supplement: Supplemental Material [file supp_gr.234435.118_Supplemental_Code.zip › Supplemental_Code/code/measures/TPTED/Table0/ground/simNo_1-n_100-m_40-s_4-minVAF_0.05-cov_2000-k_0.pdf]
